# CCN1 Promotes Mesenchymal Phenotype Transition Through Activating NF‐κB Signaling Pathway Regulated by S100A8 in Glioma Stem Cells

**DOI:** 10.1111/cns.70128

**Published:** 2024-12-11

**Authors:** Xing Guo, Shuhua Guo, Feng Tian, Zijie Gao, Yang Fan, Chuanxin Wang, Shuo Xu

**Affiliations:** ^1^ Department of Neurosurgery Qilu Hospital, Cheeloo College of Medicine and Institute of Brain and Brain‐Inspired Science, Shandong University Jinan Shandong China; ^2^ Department of Clinical Laboratory The Second Hospital of Shandong University Jinan Shandong China; ^3^ Department of Neurosurgery The First Affiliated Hospital of Shandong First Medical University &Shandong Provincial Qianfoshan Hospital Jinan Shandong China

**Keywords:** CCN1, glioblastoma, glioma stem cells, mesenchymal phenotype transition, S100A8

## Abstract

**Background:**

The presence of glioma stem cells (GSCs) and the occurrence of mesenchymal phenotype transition contribute to the miserable prognosis of glioblastoma (GBM). Cellular communication network factor 1 (CCN1) is upregulated within various malignancies and associated with cancer development and progression, while the implications of CCN1 in the phenotype transition and tumorigenicity of GSCs remain unclear.

**Methods:**

Data for bioinformatic analysis were obtained from The Cancer Genome Atlas (TCGA) and Chinese Glioma Genome Atlas (CGGA) databases. A range of primary GBM and GSC cell models were then used to demonstrate the regulatory role of CCN1 via the phenotype validation, tumor sphere formation assays, extreme limiting dilution assays (ELDA), and transwell assays. To screen out the downstream signaling pathway, we employed high‐throughput RNA‐seq. Intracranial xenograft GSC mouse models were used to investigate the role of CCN1 in vivo.

**Results:**

Among the CCN family members, CCN1 was highly expressed in MES‐GBM/GSCs and was correlated with a poor prognosis. Both in vitro and in vivo assays indicated that knockdown of CCN1 in MES‐GSCs reduced the tumor stemness, proliferation, invasion, and tumorigenicity, whereas CCN1 overexpression in PN‐GSCs exhibited the opposite effects. Mechanistically, CCN1 triggered the FAK/STAT3 signaling in autocrine and paracrine manners to upregulate the expression of S100A8. Knockdown of S100A8 inactivated NF‐κB/p65 pathway and significantly suppressed the tumorigenesis of MES‐GSCs.

**Conclusion:**

Our findings reveal that CCN1 may be an important factor in the enhanced invasiveness and MES phenotype transition of GSCs and highlight the potential to target CCN1 for treating GBM.

## Graphical abstract

CCN1‐S100A8 pathway is important for the MES phenotype transition. FAK/STAT3 and NF‐κB/p65 signaling participated in CCN1/S100A8‐mediated MES phenotype transition in GSCs.

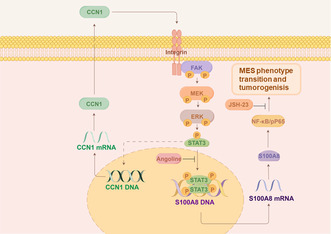

## 1 Introduction

As the most common primary brain tumor, glioblastoma (GBM) is notorious for its aggressive progression and poor prognosis [1, 2, 3]. Despite the comprehensive therapeutic efforts, the median survival for patients with newly diagnosed GBM is still miserable, even worse than pancreatic adenocarcinoma and lung squamous‐cell carcinoma [4, 5, 6, 7, 8]. Based on the characteristic gene expression, GBM can be classified into three clinically relevant molecular subtypes: classical (CL), proneural (PN), and mesenchymal (MES) [9, 10]. Patients with MES‐GBM have the worst prognosis [11, 12, 13]. Unfortunately, the natural course of GBM can be accompanied by MES transition during treatment, partly attributed to a cell subpopulation with self‐renewal activity and multi‐lineage differentiation potential, known as glioma stem cells (GSCs) [14]. The MES phenotype transition in GSCs causes therapy resistance, immunosuppression, and inevitable recurrence of tumors [15, 16, 17]. However, key regulatory factors involved in this process remain poorly understood.

Also known as Cyr61, CCN1 is encoded and secreted into the extracellular matrix and interacts with several integrins, which is vital to cell adhesion, proliferation, migration, and differentiation both physiologically and pathologically [18, 19, 20]. CCN1 dysfunction has been observed in various cancers, including breast cancer [21], colorectal cancer [22, 23], ovarian cancer [24], and pancreatic cancer [25]. For instance, CCN1 is critical for epithelial‐mesenchymal transition (EMT) and stemness in pancreatic carcinogenesis [25]. CCN1 overexpression also diminishes anti‐PD‐1 therapy responsiveness in colorectal adenocarcinoma [26]. Interestingly, CCN1 expression is distinctly upregulated in GBM tissues than in adjacent non‐tumor tissues [27]. However, available research does not address the pathological implication of CCN1 upregulation in GBM, let alone whether CCN1 is involved in the malignant phenotype transition of GSCs.

In this study, we identified that CCN1 expression is strongly associated with MES‐GBM/GSCs and unfavorable prognosis. CCN1 knockdown in MES‐GSCs reduced the tumor stemness, invasion, and tumorigenicity, whereas CCN1 overexpression in PN‐GSCs exhibited the opposite effects in vivo and in vitro. On the mechanism, CCN1 dysregulation and corresponding FAK/STAT3 signaling activation amplify the activities of each other in a regulatory loop, thereby promoting the S100A8 upregulation and NF‐κB/p65 activation, which finally facilitated phenotype transition and malignancy of GSCs.

## 2 Methods

### 2.1 Cell Lines and Culture

All patient‐derived PN‐GSC (GSC 8–11) and MES‐GSC (GSC 20, GSC 267 and GSC 28) cell lines were kindly provided from Dr. Frederick F. Lang and Dr. Krishna P. L. Bhat (University of Texas M.D. Anderson Cancer Center, USA). Based on the genetic markers, the phenotype of GSCs in this study has been identified and widely accepted [11, 15, 17]. Briefly, GSCs was digested into single cells by Accutase (Sigma‐Aldrich, USA) and then cultured in Dulbecco's modified Eagle's medium (DMEM)/F12 (Gibco, USA) supplemented with B‐27 (Gibco), 20 ng/mL recombinant human epidermal growth factor (rhEGF, R&D Systems, USA), and 20 ng/mL basic fibroblast growth factor (rhbFGF, R&D Systems).

Human glioma cell lines U251MG and U87MG were purchased from the Chinese Academy of Sciences Cell Bank and cultured in DMEM medium (Gibco, USA) with 10% fetal bovine serum (FBS). All cell lines were cultured in a humid chamber at 37°C and containing 5% carbon dioxide and 5% oxygen.

### 2.2 Cell Transfection

Independent siRNA and plasmid for CCN1 or S100A8 and related controls were obtained from Gene Pharma (Gene Pharma, China). We then used Lipofectamine 3000 transfection kit (Thermo Fisher Scientific, USA) to transfect GSCs according to the manufacturer's protocol. RNA was extracted 24 h after transfection, and protein was extracted 48 h after transfection to verify transfection efficiency. The siRNA and plasmid sequences used in this study were listed in Supporting Information.

### 2.3 Lentiviral Vector Construction

The knockdown and overexpression of CCN1 based on lentiviral vector transfection was supported by Gene Pharma (Gene Pharma). After transfection, all cells were examined for resistance to puromycin (Sigma, USA) for 15 days at a concentration of 10 μg/mL. CCN1 knockdown or overexpression was validated by western blotting. The virus‐related sequences used in this study were listed in Supporting Information.

### 2.4 Real‐Time Quantitative PCR (qRT‐PCR)

Total RNA was extracted using the RNA fast 200 kit (Fastagen Biotechnology, China). cDNA was then synthesized using the Evo M‐MLV reverse transcription kit (Accurate Biology, China). The reaction system was prepared according to manufacturer's protocol of SYBR Green Pro Taq HS qPCR kit (Accurate Biology, China) using fluorescent quantitative PCR instrument (Applied Biosysterms, USA). The sequences of PCR primer pairs were provided in the Supporting Information.

### 2.5 Western Blotting

Cell deposits were washed with cold PBS and lyzed with RIPA containing 1% protease and phosphate inhibitors (Solarbio Life Sciences, China). After SDS‐PAGE gel electrophoresis, the proteins were transferred to polyvinylidene fluoride (PVDF) membrane and incubated at 4°C overnight with primary antibody. The detailed antibody information was provided in the Supporting Information.

### 2.6 Tumor Sphere Formation Assay

GSCs were inoculated into 6‐well plates at a density of 1000 cells per well and cultured in 1.5 mL GSCs medium for 7 days. The relative diameter of tumor spheres was recorded by an optical microscope (Olympus, Japan) [28, 29].

### 2.7 Extreme Limiting Dilution Assay (ELDA)

GSCs were inoculated into 96‐well plates with a density of 50, 100, and 500 cells per well and cultured in GSCs medium for 7 days. Spheres with diameters greater than 50 μm were counted [28, 29].

### 2.8 Transwell Assay

The transwell assay was performed as previously described [30]. Briefly, GSCs were treated with FBS‐free DMEM and applied to filters coated with or without matrigel. After 48 h, the cells that had invaded the lower chamber were photographed under a microscope and counted with Image J software.

### 2.9 In Vivo Model

We constructed GSCs cells labeled with luciferase (GSC‐luciferase) by lentivirus transfection. All animal experiments were approved by the guidelines of the Institutional Animal Care and Use Committee of Qilu Hospital of Shandong University. 4‐week‐old male BALB/c nude mice (Charles river laboratories, China) was cultured at 24°C with a 12‐h diurnal cycle to prepare for the establishment of intracranial GSCs growth model in situ. Animals with similar conditions were randomly divided into control and experimental groups. After dissociation with Accutase solution, 1 × 10^6^ GSC‐luciferase cells were injected into the right frontal lobe of mice at stereotactic coordinates AP: +1.0 mm, ML: +2.5 mm, and DV: ‐3.0 mm relative to bregma [15, 31]. After intraperitoneal injection of 150 mg/kg fluorescein, the progression of tumorigenesis in vivo was measured by bioluminescence, detected, and imaged with the IVIS Lumina Series III in vitro imaging system (PerkinElmer, USA). We euthanized mice when they showed severe nervous systematic symptoms or became moribund. The survival data were recorded.

### 2.10 Bioinformatic Analysis

The gene expression and GBM patients' clinical data were extracted from The Cancer Genome Atlas (TCGA) in the HG‐U133A platform and the Chinese Glioma Genome Atlas (CGGA) in RNA‐seq platform by GlioVes data platform (http://gliovis.bioinfo.cnio.es/). Gene collection enrichment analysis (GSEA, http://www.broadinstitute.org/gsea/index.jsp) was used to detect gene sets on signaling pathways that show statistically significant differences between high‐expression and low‐expression groups of CCN1 or S100A8. The functional relationships between CCN1 and other genes were tested by two‐sided Pearson's product–moment correlation.

### 2.11 Single‐Cell RNA Sequencing Analysis

Single cell RNA sequencing (scRNA‐seq) data (GSE131928) was downloaded from Gene Expression Omnibus (GEO, https://www.ncbi.nlm.nih.gov/geo/) and analyzed using R package “Seurat 4.1.0.” Method “UMAP” was applied for the visualization of different cell clusters. Using R software package “irGSEA,” the enrichment fraction of Verhaak_GBM_MES signature is calculated and visualized by “Ucell” method.

### 2.12 Transcriptomic RNA‐Seq

Total RNA was extracted from CCN1 knockdown GSC 267, CCN1 overexpression GSC 8–11, and corresponding control GSCs using Trizol reagent (Invitrogen, USA). RNA sequencing analysis was performed using Illumina HiSeq2000 system (Illumina, USA). The sequencing results were compared with the human reference genome (GRCh38.108) and normalized using the trimmed mean of *M* values (TMM) algorithm. The edgeR software package was used to analyze the gene expression difference between samples and groups. After calculating the *p* value, multiple hypothesis testing and correction were performed to determine the *p* value threshold. According to the FPKM value, the differential expression multiple (FC) was calculated and expressed by log2 (FC). The screening criteria for differential genes were: *p* value < 0.05 and FC2 (at the same time, any group of FPKM > 1). Cluster analysis was further performed and heatmaps were generated to visualize the differentially expressed genes.

### 2.13 Flow Cytometry

Both suspended and adherent GBM cells were obtained for apoptosis analysis after treating with TMZ or DMSO (solvent control of TMZ) for 48h. Annexin V‐FITC and PI staining (BD Biosciences, USA) was leveraged for apoptosis analysis according to the instruction. The number of cells were counted by BD Accuri C6 fow cytometer.

### 2.14 Statistical Analysis

GraphPad Prism 9.5.1 was used to analyze the experimental data. Shapiro–Wilk normality test was used to evaluate the normal distribution of the data, and the data conforming to the normal distribution was presented as the mean ± SD, and the comparison between the two groups was performed by two‐independent sample *t*‐Test. Data that did not conform to normal distribution were compared between the two groups using Mann–Whitney *U* test. The correlation between different groups was evaluated by Pearson correlation algorithm. Survival was assessed by Kaplan–Meier (KM) curve and log‐rank test. *p* < 0.05 was considered to indicate statistical significance. All experiments were repeated at least three times. *p* values were indicated as follows: *p < 0.05, ** *p* < 0.01, *** *p* < 0.001.

## 3 Results

### 3.1 CCN1 Upregulation in MES‐GBM and MES‐GSCs

**FIGURE 1:**
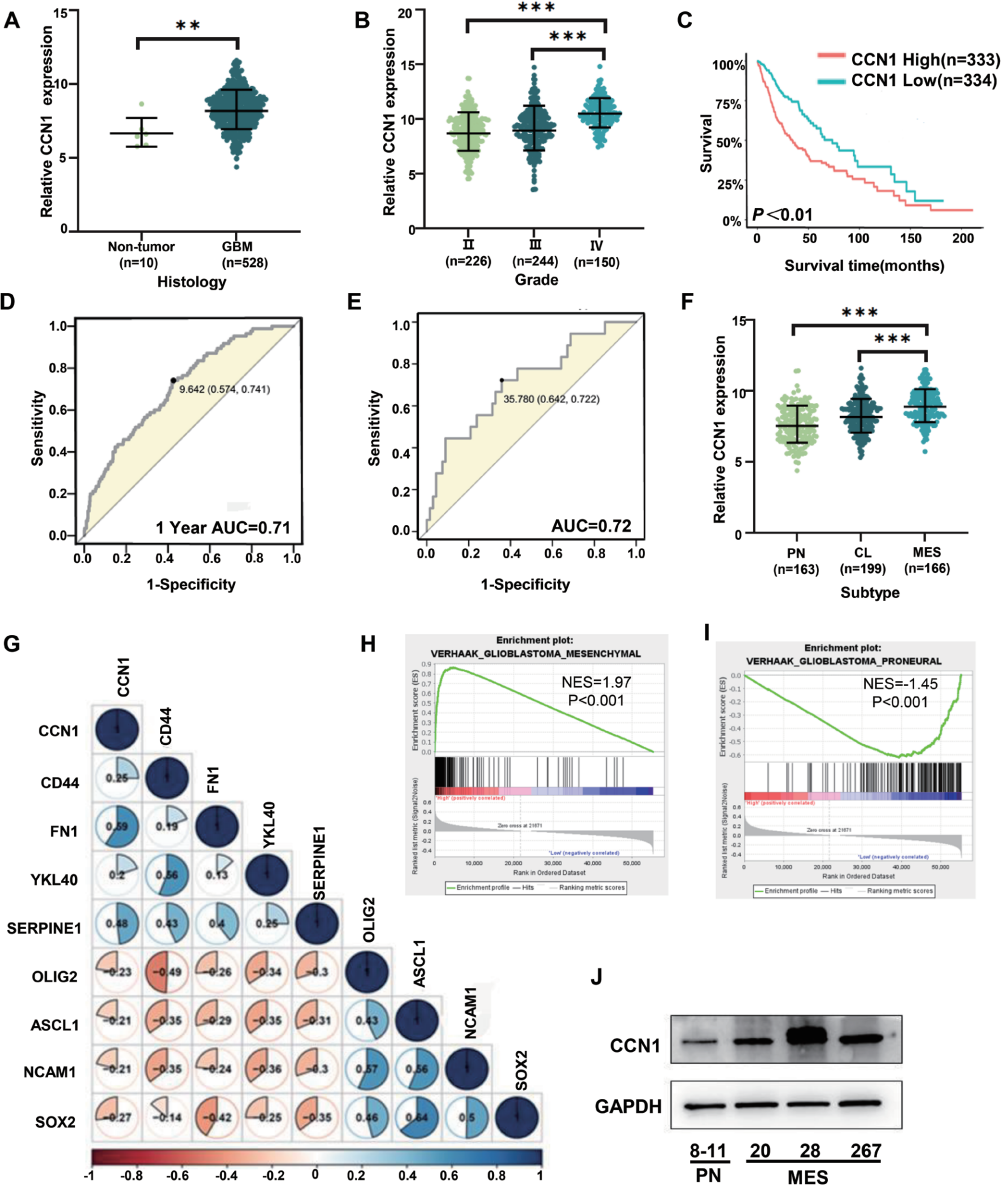
CCN1 upregulation in MES‐GBM and MES‐GSCs. (A, B) The mRNA expression of CCN1 was shown according to GBM or Non‐tumor and WHO grades in the TCGA datasets. (C) Kaplan–Meier analysis of patients with glioma with high CCN1 expression versus low CCN1 expression in the TCGA datasets. (D) Accuracy of 1‐year survival prediction of GBM patients by CCN1 expression in the TCGA datasets. (E) The accuracy of predicting PN and MES phenotypes in GBM patients by CCN1 expression in the TCGA datasets. (F) The mRNA expression of CCN1 in different phenotypes of GBM patients in the TCGA datasets. (G) The correlation of CCN1 expression with PN‐associated (OLIG2, ASCL1, NCAM1, and SOX2) and MES‐associated genes (CD44, FN1, YKL40, and SERPINE1). (H, I) Gene collection enrichment analysis (GSEA) also showed that CCN1 was positively correlated with MES phenotype, but not with PN phenotype. (J) The expression of CCN1 in different molecular subtypes of GSCs was detected by western blotting. *p < 0.05, **p < 0.01, ***p < 0.001.

To investigate the expression of CCN genes in GBM tissues, RNA‐seq data of GBM patients was acquired from TCGA database and analyzed. Among all six family members (CCN1‐6) (Figure 1A–C and Figure S1A–O), CCN1 and CCN2 were not only upregulated in GBM but also associated with the histopathologic grades (Figure 1A,B and Figure S1A,B). Compared to CCN2, high CCN1 expression was associated with shorter overall survival in patients (Figure 1C and Figure S1C). For CCN1, the areas under the curve (AUC) of the receiver operating characteristic (ROC) curve were 0.7126 for 1‐year survival (Figure 1D), indicating the prognostic accuracy of CCN1 for GBM. Similar results were demonstrated using RNA‐seq data acquired from CGGA database (Figure S2A–C). Interestingly, the AUC was 0.7156 for CCN1 to distinguish MES‐GBM from PN‐GBM (Figure 1E). Further analysis confirmed that CCN1 expression was elevated in MES‐GBM compared with CL‐MES and PN‐MES subtypes (Figure 1F and Figure S2B). At the same time, we found a significant positive correlation between the expression of CCN1 and the expression of MES subtype‐related genes (CD44, FN1, YKL40, SERPINE1) in the TCGA dataset, whereas genes associated with the PN subtype (OLIG2, ASCL1, NCAM1, SOX2) were negatively correlated (Figure 1G). Subsequently, we performed a GSEA analysis of the relationship between CCN1 and MES or PN subtype based on the TCGA dataset. The results showed that enrichment of the MES subtype was present at high CCN1 expression group, whereas enrichment of the PN subtype was present at low CCN1 expression group (Figure 1H,I). Single‐cell RNA sequencing also confirmed the correlation between CCN1 and MES subtype (Figure S2D–F).

To validate the correlation between CCN1 and MES‐GBM, we examined the CCN1 expression in a range of GSC cell lines. Intriguingly, CCN1 expression in MES‐GSC cell lines 20, 28, and 267 was elevated on both mRNA and protein levels compared with PN‐GSC cell line 8–11 (Figure 1J and Figure S2G). Collectively, these data implied that CCN1 was preferentially expressed in MES‐GBM and MES‐GSCs, which also exhibited diagnostic and prognostic potentials.

### 3.2 CCN1 Knockdown Inhibited Self‐Renewal and Invasion of MES‐GSCs

**FIGURE 2:**
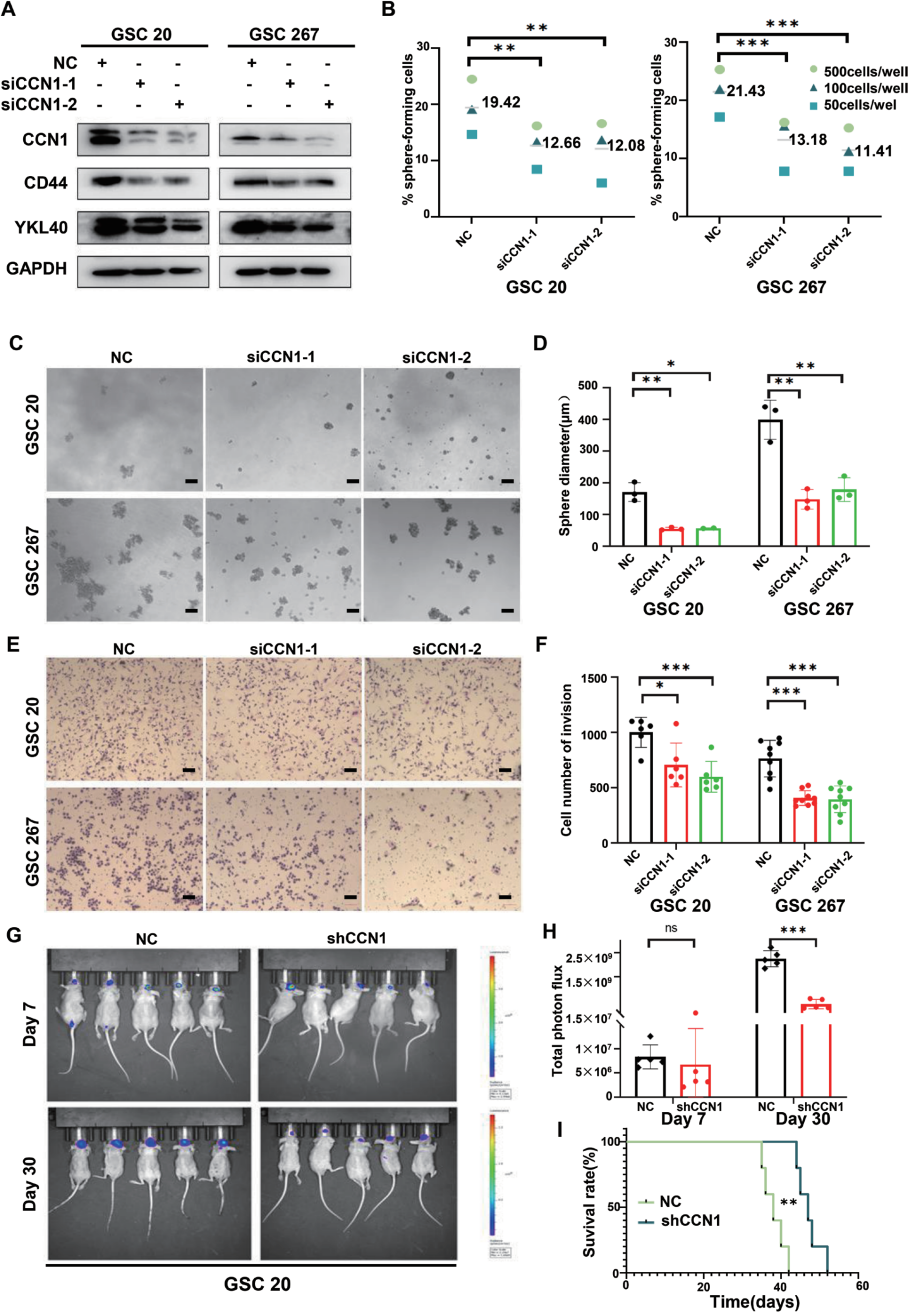
CCN1 knockdown inhibited self‐renewal and invasion of MES‐GSCs. (A) The expression of CCN1 and MES phenotype markers in GSC 20 and GSC 267 after CCN1 knockdown were measured by western blotting. (B–D) Extreme limiting dilution assay and tumor sphere formation assay showed that the tumor formation rates decreased after CCN1 knockdown. Scale bar = 100 μm. (E, F) Transwell assay showed the invasion of GSC 20 and GSC 267 after CCN1 knockdown. Scale bar = 100 μm. (G, H) The quantification of the photon counts of GSC 20 xenografts. The tumor sizes were monitored on day 7 and day 30. (I) Kaplan–Meier curves showed the survival of GSC 20 xenograft‐bearing mice in the different groups. *p < 0.05, **p < 0.01, ***p < 0.001.

To investigate the role of CCN1 in the aggressive behavior of MES‐GSCs, two independent siRNA sequences were employed to knockdown CCN1 in GSC 20 and GSC 267, which had high basal CCN1 levels (Figure 2A and Figure S2H). Unsurprisingly, CCN1 knockdown resulted in the downregulations of CD44 and YKL40, two well‐defined MES phenotype markers in GSCs and GBM cell lines (Figure 2A and Supplementary Fig. S5A). Sphere diameter and sphere formation ability of GSC 20 and GSC 267 were also markedly reduced after the CCN1 knockdown, which implied the decreased self‐renewal ability in MES‐GSCs (Figure 2B–D). The migration and invasion abilities of MES‐GSCs were also significantly inhibited, as shown in Figure 2E,F and Supplementary Figure S2I–J and 5C. Furthermore, we constructed xenograft mouse models to demonstrate the consequences of CCN1 silencing in vivo. GSCs transfected with shRNAs targeting CCN1 or control sequence were implanted in situ into the brains of nude mice. Compared with the control mice, CCN1‐silencing mice exhibited decreased tumor burdens on Day 30 and prolonged survival periods (Figure 2G–I). These results demonstrated that silencing of CCN1 in the MES‐GSCs reduced self‐renewal, invasion, and tumorigenicity both in vitro and in vivo.

### 3.3 CCN1 Overexpression Promotes Tumor Growth and Phenotype Transition of PN‐GSCs

**FIGURE 3:**
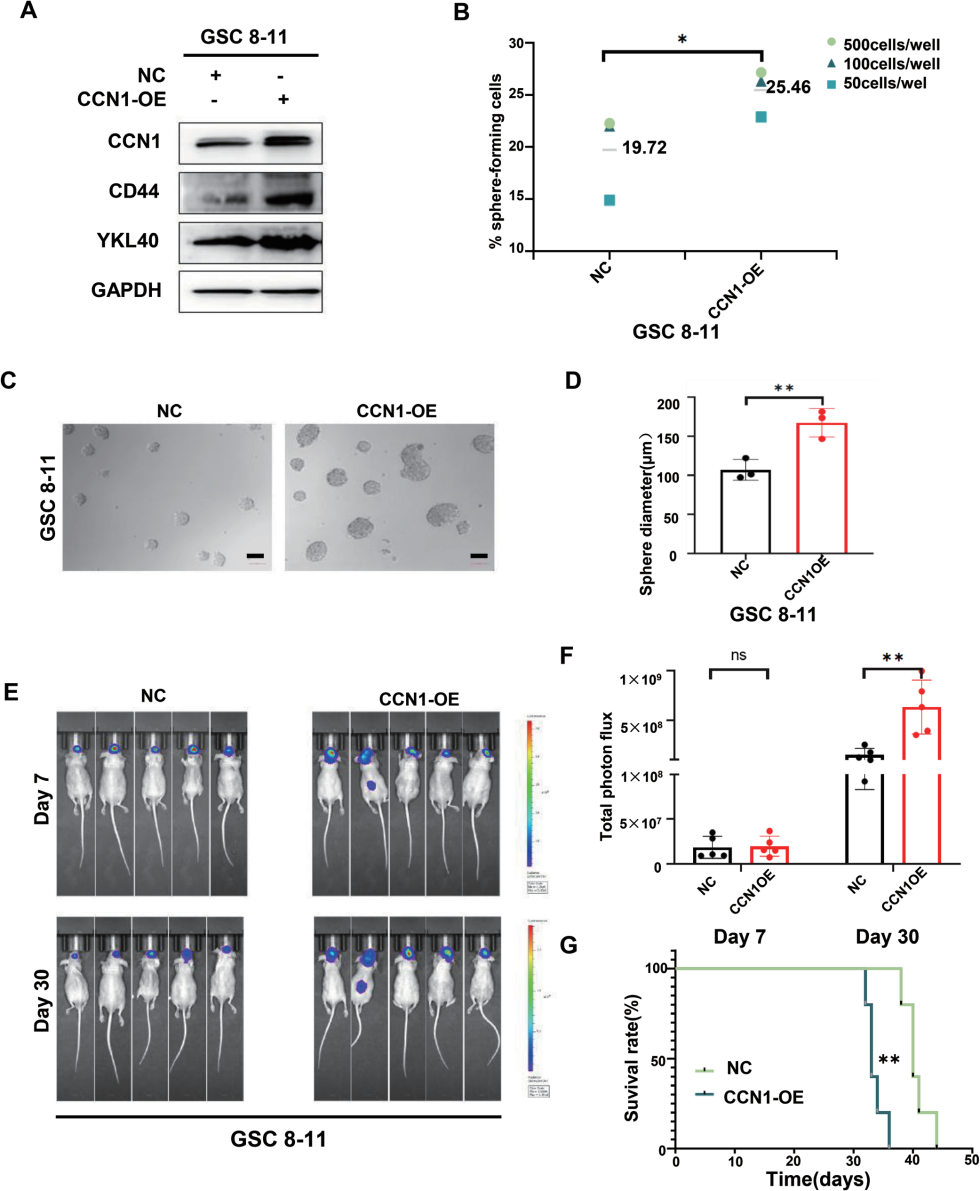
CCN1 overexpression promotes tumor growth and phenotype transition of PN‐GSCs. (A) The expression of CCN1 and MES phenotype markers in GSC 8–11 were measured by western blotting. (B–D) Extreme limiting dilution assay and tumor sphere formation assay showed that the tumor formation rates increased after CCN1 overexpression. Scale bar = 100 μm. (E, F) The quantification of the photon counts of GSC 8–11 xenografts. The tumor sizes were monitored on day 7 and day 30. (G) Kaplan–Meier curves showed the survival of GSC 8–11 xenograft‐bearing mice in the different groups. *p < 0.05, **p < 0.01, ***p < 0.001.

Considering the anti‐tumor effects of CCN1 silencing in the MES‐GSCs, we overexpressed CCN1 in the PN‐GSC 8–11 to further confirm its pathophysiological role. As shown in Figure 3A and Supplementary Fig. S5B, CCN1 overexpression promoted the expression of CD44 and YKL40 in PN‐GSCs and GBM cell lines, indicating the MES differentiation. Both ELDA and sphere‐forming assays demonstrated a marked increase in sphere size and sphere‐ formation ability (Figure 3B–D). Moreover, CCN1 overexpression in vivo significantly enhanced the intracranial tumor burdens of GSC 8–11 on Day 30 (Figure 3E,F) with diminished survival (Figure 3G). In summary, these results indicated that CCN1 overexpression promoted the progression and phenotype transition in PN‐GSCs.

### 3.4 CCN1 Regulates the Expression of S100A8 via FAK‐STAT3 Signaling

**FIGURE 4:**
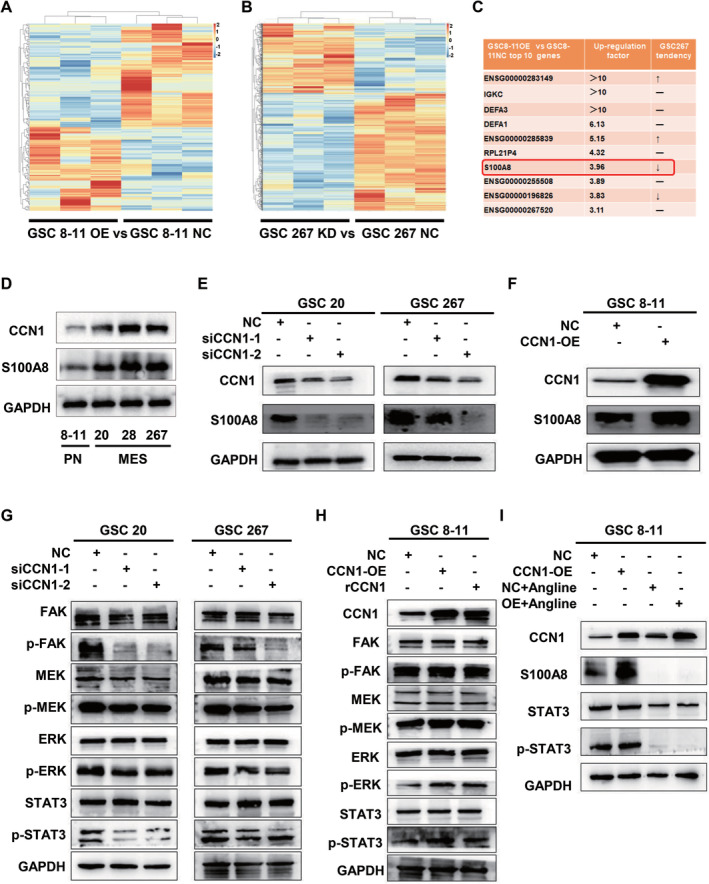
CCN1 regulates the expression of S100A8 via FAK‐STAT3 signaling. (A) The heatmap of transcriptomic RNA‐seq results of overexpressing CCN1 group and control group in GSC 8–11. (B) The heatmap of the distribution of gene differences between GSC 267 knockdown CCN1 group and GSC 267 control group. (C) The top 10 upregulated genes in GSC 8–11 with CCN1 overexpression were cross‐compared with the downregulated genes in GSC 267 with CCN1 silencing. (D) Western blotting showed that S100A8 was expressed in GSCs of different molecular subtypes. (E, F) The effect of CCN1 knockdown or overexpression on the expression of S100A8 was examined. (G, H) The effect of CCN1 knockdown or overexpression on the expression of FAK/MEK/ERK signaling pathway proteins were examined. (I) Angoline treatment inhibited the proteins expression of S100A8, STAT3, and p‐STAT3 in CCN1‐overexpressed GSC 8–11. *p < 0.05, **p < 0.01, ***p < 0.001.

To understand the regulatory mechanism of CCN1 on GSCs, transcriptomic RNA‐seq assays were conducted (Figure 4A,B). The top upregulated genes in GSC 8–11 with CCN1 overexpression were enlisted and then crosschecked with the downregulated genes in GSC 267 with CCN1 silencing (Figure 4C). Based on the bioinformatics analyses of TCGA dataset, we focused on S100A8 among the candidate genes that met the above criterion. Specifically, S100A8 was upregulated with the histopathologic grades of gliomas, primarily abundant in GBM and MES‐GBM subtype (Figure S3A–C). High S100A8 expression was associated with shorter survival in GBM patients (Figure S3D). Moreover, the correlation between CCN1 and S100A8 was confirmed in GBM (Figure S3E). In addition, we also analyzed the expression of genes related to PN and MES subtypes after knockdown or overexpression of CCN1, and the results further proved that CCN1 and MES phenotypes were positively correlated (Figure S3G–H).

As shown in Figure 4D and Figure S3F, we observed elevated S100A8 expression in MES‐GSC 20, 28, and 267 rather than PN‐GSC 8–11. The expression of S100A8 was remarkedly inhibited in GSC 20 and 267 with CCN1 silencing, while increased in GSC 8–11 after CCN1 overexpression with the same trend in GBM cell lines (Figure 4E,F and Supplementary Fig. S6A‐B). Previous studies have shown that CCN1 activates the FAK/MEK/ERK signaling pathway in colorectal cancer by binding to integrin αVβ5 to activate STAT3, a key upstream molecule known to regulate S100A8 [32, 33, 34]. Based on the above evidence, we hypothesized that CCN1 regulates S100A8 expression in GSCs by activating the FAK/MEK/STAT3 signaling pathway. To validate this hypothesis, we found that p‐FAK/p‐MEK/p‐ERK/p‐STAT3 were significantly decreased in GSC 20 and 267 with CCN1 silencing (Figure 4G), whereas markedly elevated in GSC 8–11 with CCN1 overexpression (Figure 4H). Interestingly, p‐FAK/p‐MEK/p‐ERK/p‐STAT3 also significantly increased after the addition of exogenous recombinant CCN1, implicating an autocrine/paracrine regulatory mechanism of this secreted protein within the tumor microenvironment (Figure 4H). In the GBM cell line, we also demonstrated that CCN1 regulates the expression of S100A8 via FAK‐STAT3 signaling (Supplementary Fig. S6C‐D). In addition, angoline (50 μM), a potent and selective STAT3 inhibitor, could attenuate the S100A8 upregulation induced by CCN1 overexpression (Figure 4I).

We also synthesized two antisense oligonucleotides (ASO) as small molecule inhibitors to target CCN1. The results showed that inhibition of CCN1 significantly reduced S100A8 and downstream key signaling pathways and MES phenotypic markers (Supplementary Fig. S7A‐C).

### 3.5 S100A8 Regulates the Growth, Invasion, and MES Phenotype Transition of GSCs

**FIGURE 5:**
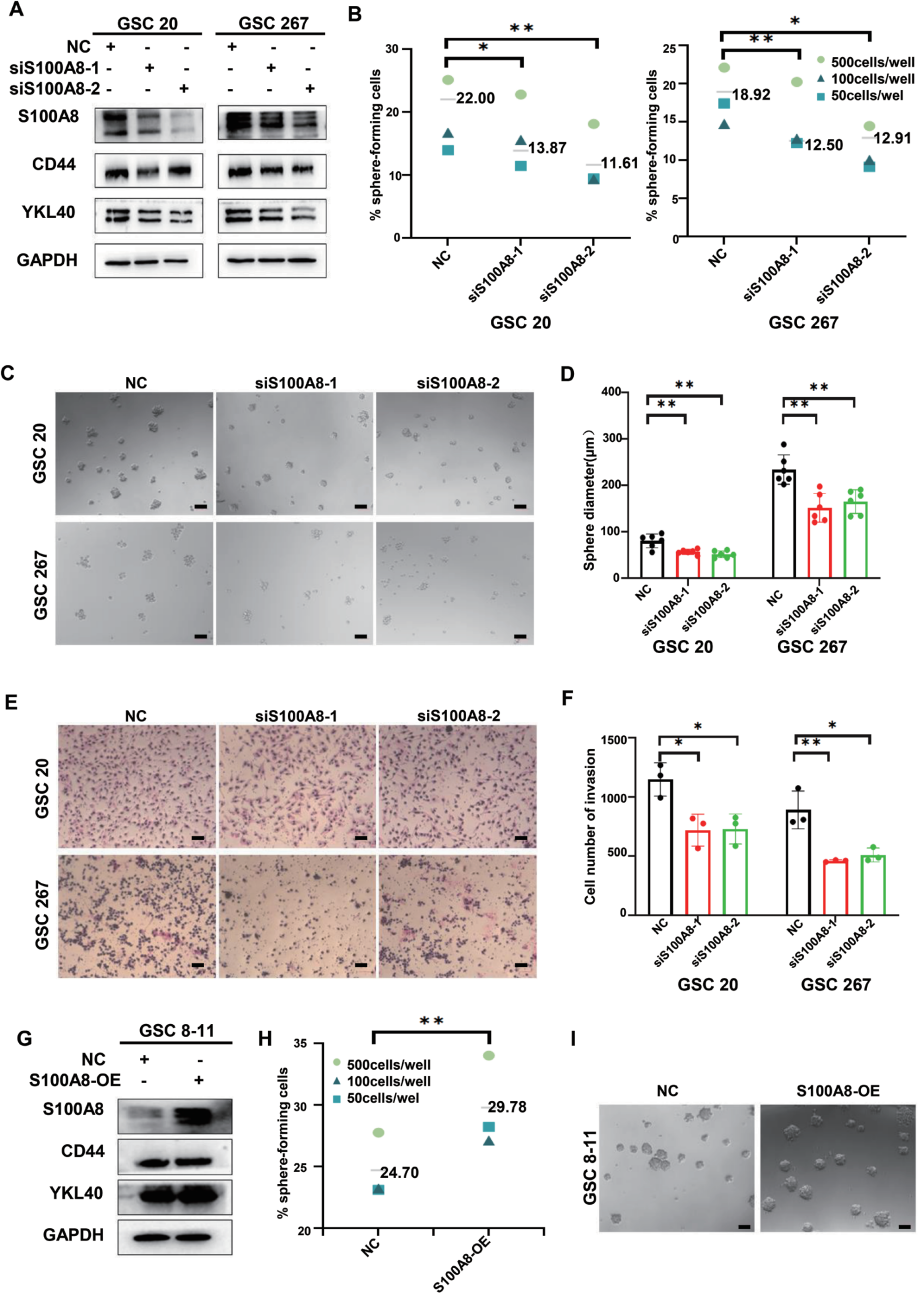
S100A8 regulates the growth, invasion and MES phenotype transition of GSCs. (A) The expression of S100A8 and MES phenotype markers in GSC 20 and GSC 267 after S100A8 knockdown were measured by western blotting. (B–D) Extreme limiting dilution assay and tumor sphere formation assay showed that the tumor formation rates decreased after S100A8 knockdown. Scale bar = 100 μm. (E, F) Transwell assay showed the invasion of GSC 20 and GSC 267 after S100A8 knockdown. Scale bar = 100 μm. (G) The expression of S100A8 and MES phenotype markers in GSC 8–11 after S100A8 overexpression were measured by western blotting. (H, I) Extreme limiting dilution assay and tumor sphere formation assay showed that the tumor formation rates increased after S100A8 overexpression. Scale bar = 100 μm. *p < 0.05, **p < 0.01, ***p < 0.001.

Mainly located in cytoplasm and nucleus, S100A8 can trigger multiple signal transduction pathways to mediate microtubule constitution and pathogen defense, as well as intricate cancer growth, metastasis, drug resistance, and prognosis [35, 36]. To investigate the role of S100A8 in GSC self‐renewal and tumor promotion, we transfected GSC 20 and GSC 267 with two independent siRNA sequences separately to silence the S100A8 expression. Western blotting and qPCR assays were used to verify the knockdown efficiency (Figure 5A and Figure S4A). As expected, CD44 and YKL40 expressions were inhibited with the S100A8 knockdown in GSCs and GBM cell lines (Figure 5A and Supplementary Fig. S8A). Sphere formation ability and sphere diameter also decreased, which indicated the impaired self‐renewal ability of GSCs (Figure 5B–D). Consistent with CCN1, the migration and invasion ability of GSCs were significantly inhibited after S100A8 knockdown (Figure 5E,F and Supplementary Figure S4B,C and S8C). Based on the potential role of S100A8 in drug resistance, we treated different groups of GSC with TMZ and performed apoptosis assays. The results showed that inhibition of S100A8 significantly increased the sensitivity of glioma cells to TMZ treatment, which further validated the important role of S100A8 in GBM for therapeutic resistance (Supplementary Fig. S8D).

In contrast, PN‐GSC 8–11 was transfected with the overexpression plasmid to demonstrate the tumorigenicity of S100A8 (Figure 5G). Unsurprisingly, CD44 and YKL40 expression increased after the S100A8 overexpression in GSCs and GBM cell lines (Figure 5G and Supplementary Fig. S8B). In addition, the self‐renewal ability was significantly enhanced with the increase of S100A8 level, as shown in the ELDA and sphere‐forming assays (Figure 5H,I). Taken together, these results indicated that S100A8 modulated the growth, invasion, and MES phenotype transition of GSCs.

### 3.6 CCN1/S100A8 Regulates NF‐κB Signaling Pathway Activity

**FIGURE 6:**
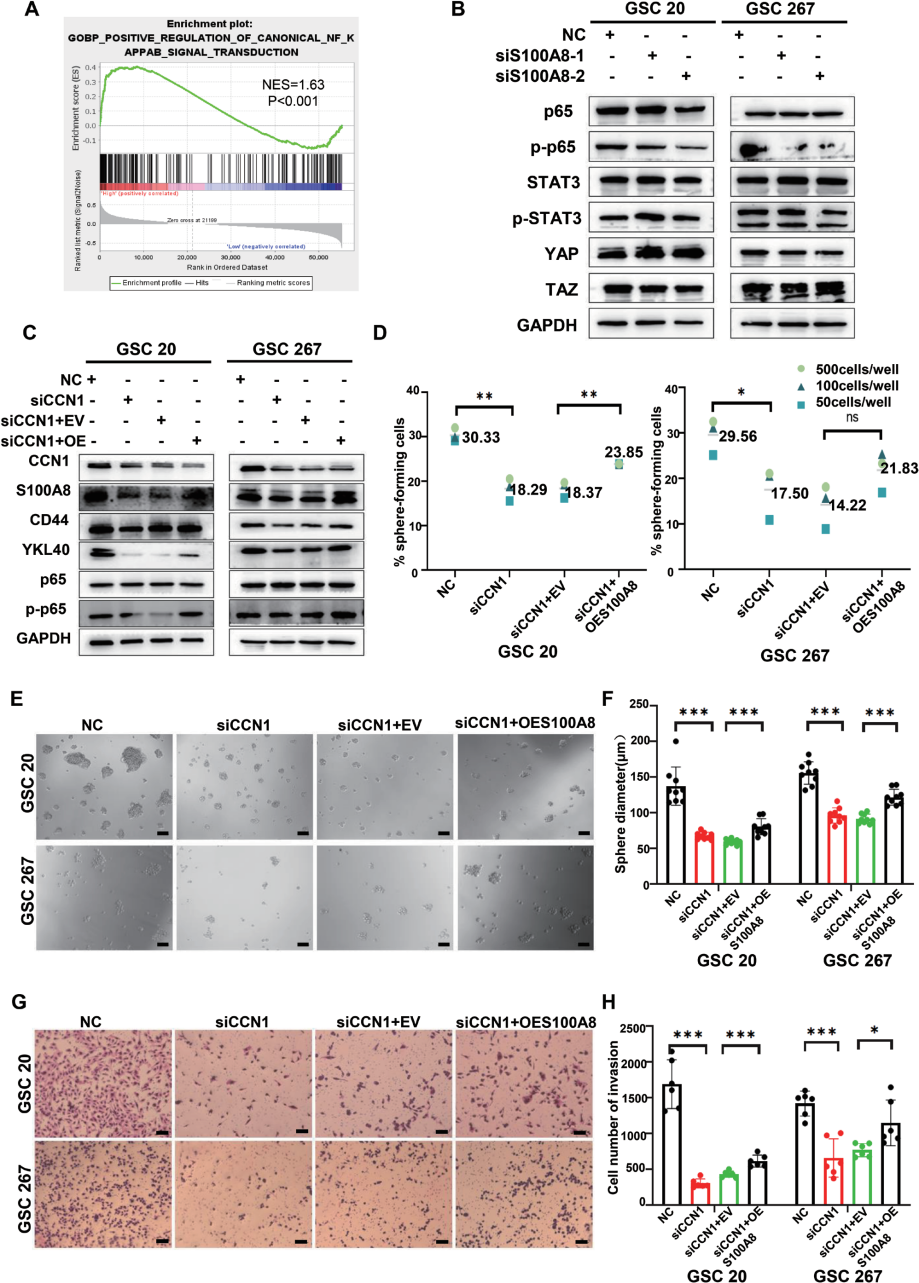
CCN1/S100A8 regulates NF‐κB signaling pathway activity. (A) Gene collection enrichment analysis (GSEA) showed that high expression of S100A8 was positively correlated with enhanced expression of NF‐κB/p65 pathway in the TCGA datasets. (B) The expression of MES phenotype transition associated signaling pathway proteins in GSC 20 and GSC 267 after S100A8 knockdown were measured by western blotting. (C) Rescuing S100A8 in CCN1‐silenced GSCs restores the expression of CD44, YKL40 and p‐p65. (D–F) Extreme limiting dilution assay and tumor sphere formation assay showed that re‐expression of S100A8 saved neuroglobular growth compared with empty carrier controls with CCN1 silencing. Scale bar = 100 μm. (G, H) Transwell assay showed that the invasion ability of GSC 20 and GSC 267 was restored after S100A8 re‐expression. Scale bar = 100 μm. *p < 0.05, **p < 0.01, ***p < 0.001.

To better understand the regulatory mechanism of CCN1/S100A8, we performed the GSEA analysis to evaluate the interaction between S100A8 and multiple key MES phenotype transition associated signaling pathways, including STAT3, YAP, and NF‐κB, etc. Interestingly, the NF‐κB signaling pathway was significantly enriched in the high expression group of S100A8 (Figure 6A). Further western blotting demonstrated that p65, rather than STAT3 or YAP, was downregulated in the MES‐GSC 20 and 267 with S100A8 silencing (Figure 6B).

Next, we re‐expressed S100A8 in CCN1‐silenced MES‐GSC 20 and 267(Figure 6C). As expected, S100A8 rescue partially, yet not wholly, restored the MES marker expression and self‐renewal capacity inhibited by CCN1 knockdown, as shown in the ELDA and sphere‐forming assays (Figure 6C–F). Notably, the p‐p65 level was downregulated after CCN1 silencing but then retained due to S100A8 rescue (Figure 6C). Meanwhile, CCN1‐knockdown‐induced inhibition of GSC migration and invasion was also aborted to some extent by re‐expression of S100A8 (Figure 6G,H and Figure S4D,E). Thus, our results demonstrated that CCN1/S100A8 promoted MES phenotype transition and tumorigenicity by affecting NF‐κB signaling.

### 3.7 NF‐κB Inhibitor Can Eliminate Malignant Progression of CCN1‐Induced GSCs

**FIGURE 7:**
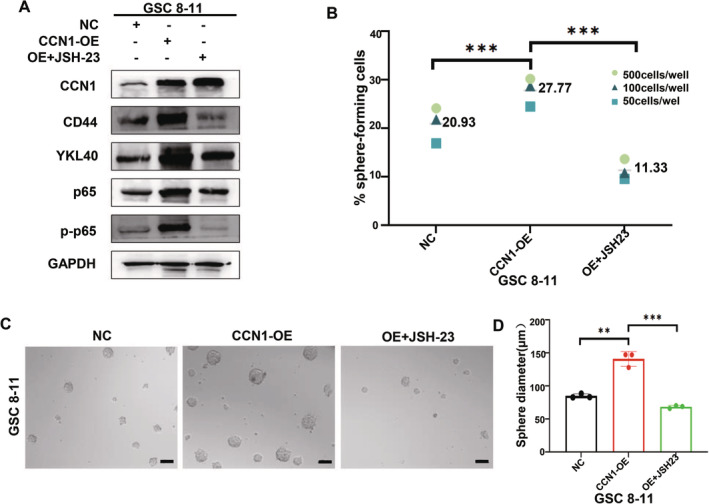
NF‐κB inhibitors can eliminate malignant progression of CCN1‐induced GSCs. (A) JSH‐23 treatment inhibited the protein expression of CD44, YKL40 and p‐p65 downstream genes in CCN1‐overexpressed GSC 8–11. (B–D) JSH‐23 treatment reduced the self‐renewal capacity of CCN1 overexpression GSC 8–11 as measured by extreme limiting dilution assay and tumor sphere formation assay. Scale bar = 100 μm. *p < 0.05, **p < 0.01, ***p < 0.001.

Based on the abovementioned evidence, we abrogated the NF‐κB signaling pathway in CCN1‐overexpressed GSC 8–11 with JSH‐23 (250 ng/mL), an NF‐κB inhibitor. Although CCN1 overexpression prompted a distinct increase in CD44, YKL40, and p‐p65 as expected, such change could be reversed by JSH‐23 treatment (Figure 7A). We further performed ELDA to explore the effect of CCN1/S100A8‐NF‐κB on tumorigenesis. We observed that NF‐κB inhibitor in CCN1‐overexpressed GSC 8–11 suppressed sphere formation capacity (Figure 7B). Similarly, JSH‐23 remarkedly reduced the sphere expansion in the neurosphere‐forming assays (Figure 7C,D).

## 4 Discussion

GBM is a highly aggressive malignancy with a poor prognosis [1]. Despite the emergence of new treatment approaches such as immunotherapy and molecular targeted therapy, the outcomes for patients with GBM remains miserable, largely due to the presence of GSCs [37, 38, 39]. GBM is a highly heterogeneous entity, and therapeutic interventions often drive the transformation of PN‐GSCs into more malignant and treatment‐resistant MES‐GSCs [16, 17, 31]. This MES phenotype transition plays a crucial role in the failure of comprehensive treatments [16, 17, 31]. Hence, it is imperative to understand the regulatory mechanisms of MES phenotype transition and maintenance in GSCs, in order to develop effectively targeted therapies for GBM treatment.

CCN family proteins are well known as stromal cell regulatory factors involved in intracellular and extracellular signaling, playing an important role in cell proliferation, differentiation, chemotaxis, adhesion, angiogenesis, and ECM formation [20]. Recently, increasing studies demonstrated that CCN family was inseparable from malignant progression of GBM [19, 27, 40]. For instance, Tao et al. [41] reported that CCN4 activated the Wnt/β‐catenin pathway, exerting a dual effect by maintaining GSCs and promoting the presence of tumor‐supportive macrophages. As the potentially carcinogenic role of CCN family in GBM, our study found that, among CCN family, CCN1 might be a novel oncogene responsible for GBM occurrence and progression via modulating the MES phenotype transition of GSCs. As for CCN1, glioma‐related research had primarily focused on its immunomodulation effect of immune cells adhension and recruitment. Uneda et al. [42] showed that differentiated GBM cells accelerated tumor progression by shaping the tumor microenvironment through CCN1‐mediated macrophage infiltration. Additionally, extracellular CCN1 limited the efficacy of oncolytic treatments in glioma by modulating macrophage activity via interacting with integrin α6β1 [43, 44]. Although previous studies had indicated CCN1 expression was upregulated in GBM tissues and associated with poor survival of individual [27], the precise mechanisms by which CCN1 regulated the progression of GBM cells themselves, especially the role and detail underlying mechanism of CCN1 in facilitating MES phenotype transition of GSCs, largely remain unknown.

In our study, we revealed that CCN1 promoted MES phenotype transition and maintenance by regulating S100 Calcium Binding Protein A8 (S100A8) in GSCs. Similar to CCN1, S100A8 was firstly reported to take part in reconstruct tumor microenvironment by recruitment of immune cells, leading to tumor growth, metastasis, and premetastatic niche formation [45, 46]. In this study, we noticed that the overexpression or knockdown of S100A8 and CCN1 could have similar effects on promoting the mesenchymal (MES) transition. Additionally, overexpression of S100A8 partially reversed the effects of CCN1 knockdown on GSC MES subtypes and malignant biological behaviors. Although the possibility that CCN1 and S100A8 affect GSC through different pathways or mechanisms cannot be ruled out, given that intervening CCN1 significantly affects the expression of S100A8, as we concluded that CCN1 affects GSC MES phenotypes and malignant biological behaviors through the regulation of S100A8. Previous studies explored the role of S100A gene family in glioma and discovered that S100A8 was highly expressed and proved to be a marker for predicting prognosis related to immune‐based score model [33, 47]. Although S100A8 had been shown to be involved in augmenting the malignant biological progression of glioma, such as proliferation, invasion, and migration, there was no specific research referring to the role of S100A8 in regulating MES phenotype transition of GSCs [48, 49]. Nevertheless, in colorectal, Xu et al. confirmed USF2/S100A8 axis promoted cell migration and invasion through modulating EMT [46]. As the potentially vital role of CCN1/S100A8 axis in MES phenotype transition of GSCs, the attempt to investigate the downstream signaling pathway regulated may provide novel intervention target for GBM treatment.

Future studies on glioma stem cell (GSC) transformation should focus on exploring in detail the molecular mechanisms underlying their plasticity, including how epigenetic modifications regulate the transition between the stem‐like and differentiated states. It is critical to study the role of metabolic reprogramming in supporting GSC survival and drug resistance. In addition, understanding the interactions between GSC and the tumor microenvironment, particularly the effects of hypoxia, immune evasion mechanisms, and extracellular matrix components, can shed light on how GSC contribute to tumor invasion, recurrence, and drug resistance. Studies of the interplay between key signaling pathways, such as STAT3 and NF‐κB, can also provide insight into the regulatory networks that drive GSC transformation, providing the possibility of new targeted therapies that disrupt these processes.

A limitation of this study is that it focuses on the use of a single PN GSC, which potentially makes the results less representative of the experiment. In addition, this study did not assess CCN1 expression in the GBM tumor microenvironment in the context of other key cell types (e.g. astrocytes, neurons, and microglia). These cell types play critical roles in tumor progression and response to therapy, and their interaction with GSCs could significantly influence the expression and function of CCN1. By not assessing CCN1 levels in these normal cell populations, the study may overlook important microenvironmental factors that contribute to GBM pathology.

In general, CCN1 regulates the biological behavior of tumors in form of extracellular matrix protein. Prior study revealed that CCN1 enhanced the migration and invasion of glioma cells dependence on binding to integrins and activating downstream of STAT3 pathway [50]. More importantly, our study identified that CCN1 regulated the expression of S100A8 through the FAK/ERK/STAT3 signaling pathway during the process of MES phenotype transition in GSCs, which can be triggered by exogenous recombinant CCN1 and interrupted by a STAT3 inhibitor, Angoline. Expanding on a previous study indicating that STAT3 acted as an upstream regulator promoting transcription of CCN1 in adipose‐derived stem cells, we proposed that a positive feedback loop exists between CCN1 and STAT3, and CCN1 may activate the FAK/ERK/STAT3 signaling pathway by binding integrins through both autocrine and paracrine manner [32]. As outlined in our proposal, a noteworthy strategy for addressing GBM involved intervening in the receptor‐adapter binding of CCN1 with integrins, warranting additional attention.

The MES phenotype transition of GSCs was primarily regulated by the NF‐κB, STAT3, and YAP pathways [16, 51, 52]. Our study confirmed that CCN1/S100A8 axis activated the NF‐κB signaling pathway during promoting MES phenotype transition of GSCs. Consistently, NF‐κB inhibitor, JSH‐23, could inhibit MES phenotype transition and maintenance of GSCs influenced by CCN1/S100A8 axis. As for CCN1, it was well known to play an important role in various diseases by activating the NF‐κB pathway. For instance, Won, Choi, and Jun [53] found that CCN1 regulated self‐renewal and differentiation of intestinal stem cells by inducing NF‐κB‐dependent Jag1 expression. Jiang et al. [54] demonstrated that CCN1 promoted autoimmune hepatitis through the PI6K/Akt/NF‐κB signaling pathway. As for S100A8, plenty of studies showed that S100A8 regulated the NF‐κB pathway in variety of biological processes. Wu et al. found that S100A8/A9 improved the apoptosis rate of microglia by up‐regulating the NF‐κB signaling pathway and played a role as a pro‐inflammatory factor [55]. Wang et al. [56] presented that down‐regulation of S100A8 inhibited the proliferation of renal cell cancer cells and promoted apoptosis by inhibiting the NF‐κB pathway. Inconsistent with our results, it is reported that overexpression of CCN1 inhibited the NF‐κB signaling pathway in pulmonary hypertension [57]. In our perspective, the varied binding and interaction of CCN1 with different integrins, dependent on cell type and environment, result in multiple downstream effects [20]. And this phenomenon may elucidate the contrasting impacts of CCN1 on the NF‐κB pathway observed between pulmonary hypertension and GBM.

In conclusion, we identified CCN1, which is significantly upregulated in MES‐GBM/GSCs and is correlated with a poor prognosis. We revealed that CCN1 as extracellular matrix protein promoted MES phenotype transition and maintenance by regulating S100A8 in GSCs. Moreover, the regulatory loop of FAK/STAT3 and NF‐κB/p65 signaling participated in CCN1/S100A8‐mediated MES phenotype transition in GSCs. Our findings expand the understanding of the heterogeneity and plasticity of GBM and provide a potential therapeutic strategy for GBM treatment.

## Author Contributions

S.X. designed the study. X.G., S.G. and F.T. performed the experimental work. Z.G. and Y.F. performed data analyses. X.G. and S.G. wrote this manuscript. X.G., S.G., F.T., Z.G., Y.F. and C.W. edited and revised the manuscript. All authors read and approved this manuscript.

## Ethics Statement

The study was authorized by the Ethics Committee of Qilu Hospital. The animal experiments in this study were approved by the Ethics Committee of Qilu Hospital (No. DWLL‐2023‐150).

## Conflicts of Interest

The authors declare no conflicts of interest.

## Supporting information


Data S1.

Data S2.

## Data Availability

Publicly available datasets were applied in this research. These resources could be found here: GlioVes data platform: http://gliovis.bioinfo.cnio.es/; Other data obtained and/or analyzed during the current study were available from the corresponding authors on reasonable request.
