# Supplementary material for: CCN1 Promotes Mesenchymal Phenotype Transition Through Activating NF‐κB Signaling Pathway Regulated by S100A8 in Glioma Stem Cells
Source: CNS Neurosci Ther. 2024 Dec 11;30(12):e70128. doi: 10.1111/cns.70128 (PMC11632201; doi:10.1111/cns.70128)

# Full unedited blots for Figure

## Figure 1J

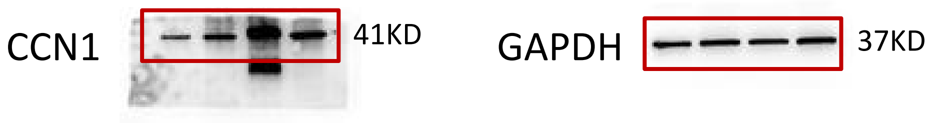


## Figure 2A

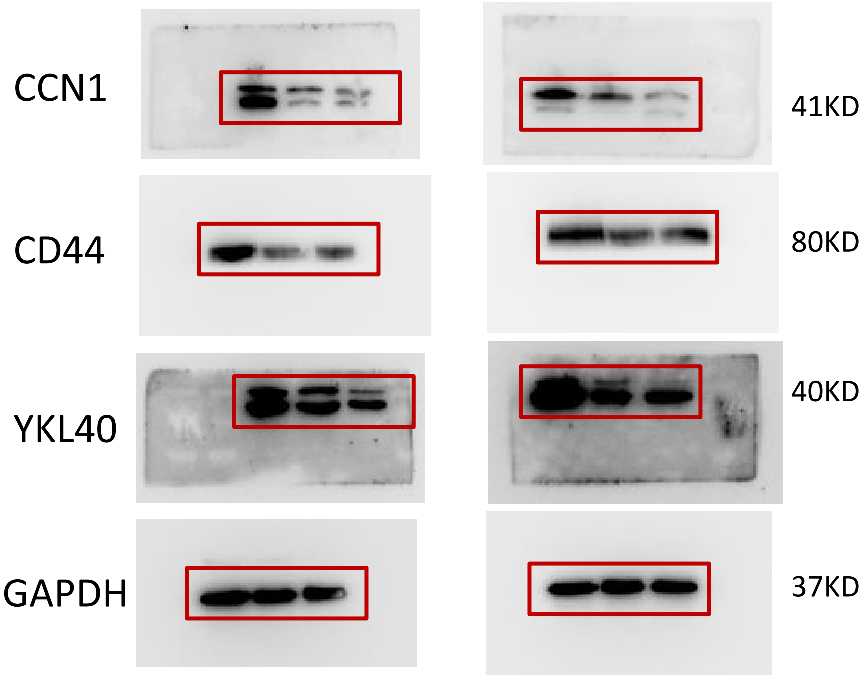


## Figure 3A

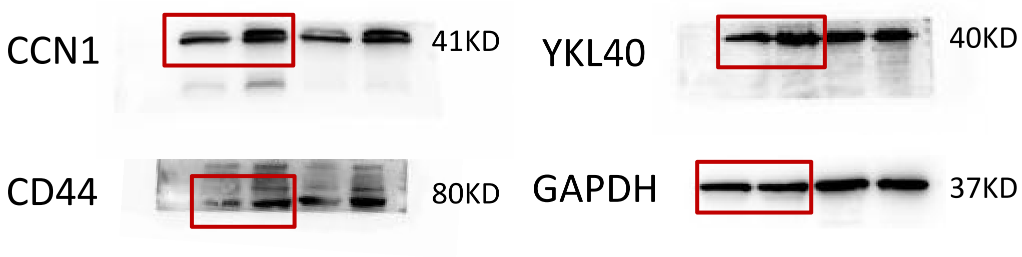


## Figure 4D

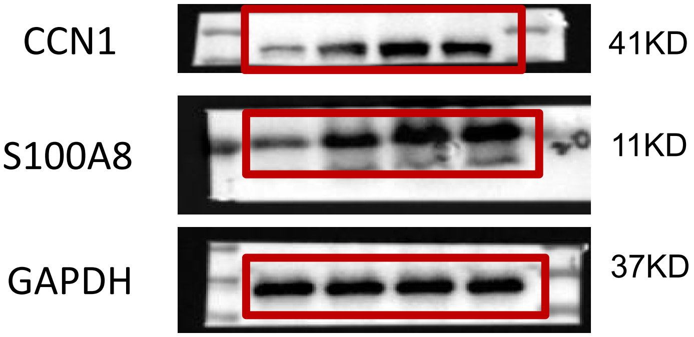

## Figure 4E

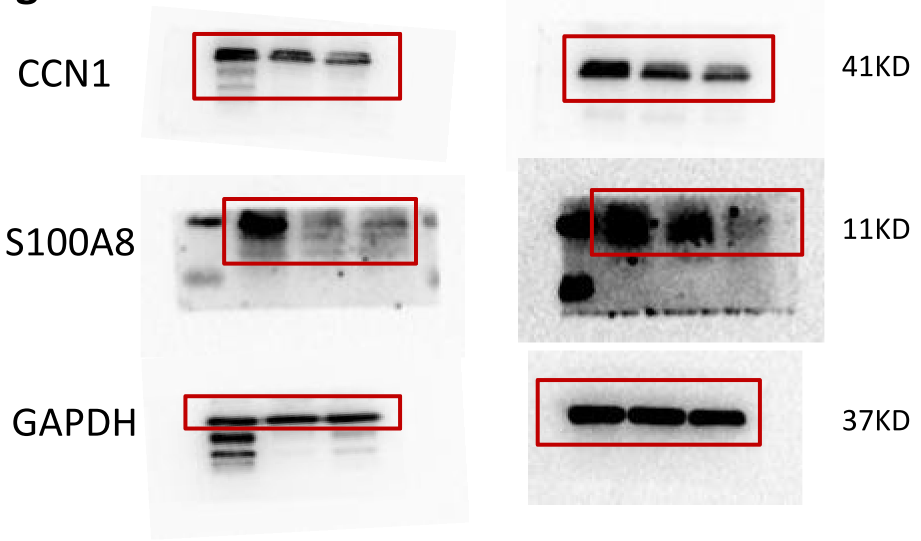


## Figure 4F

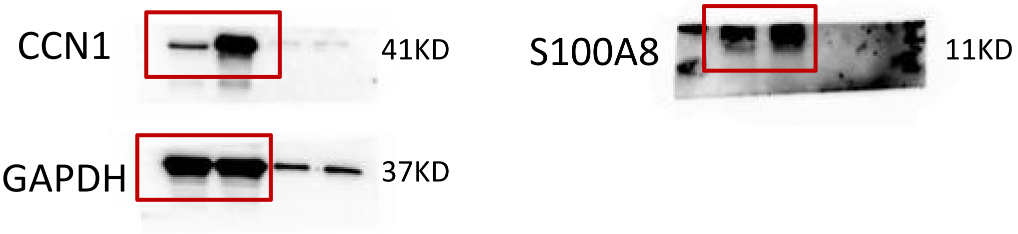


## Figure 4G

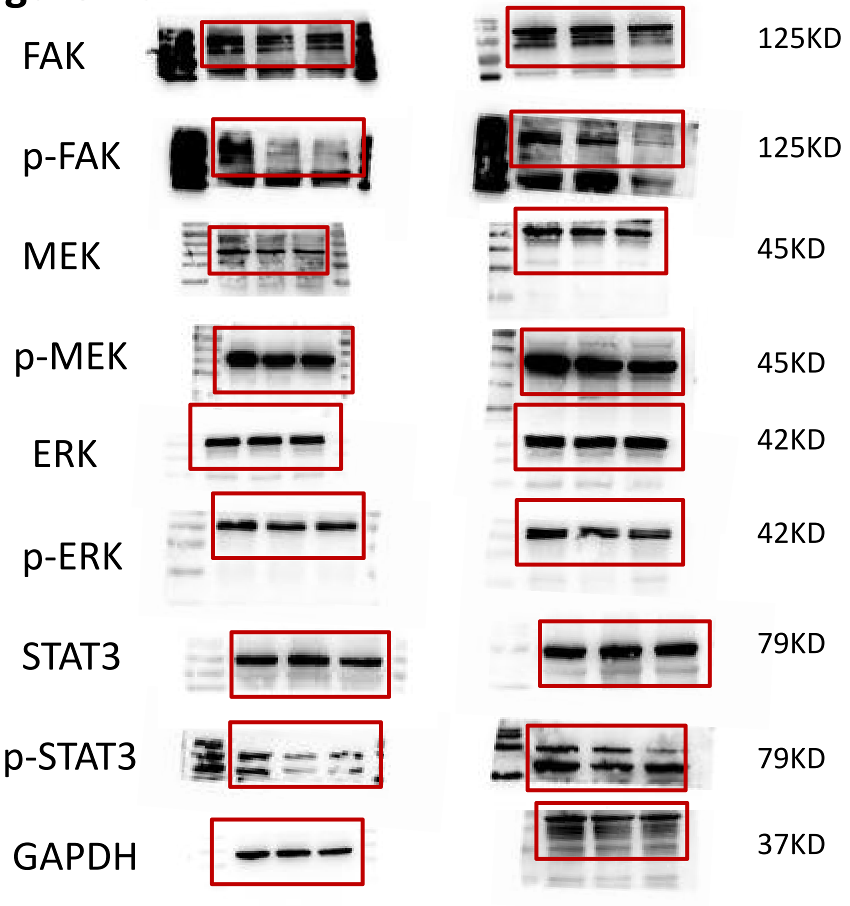

## Figure 4H

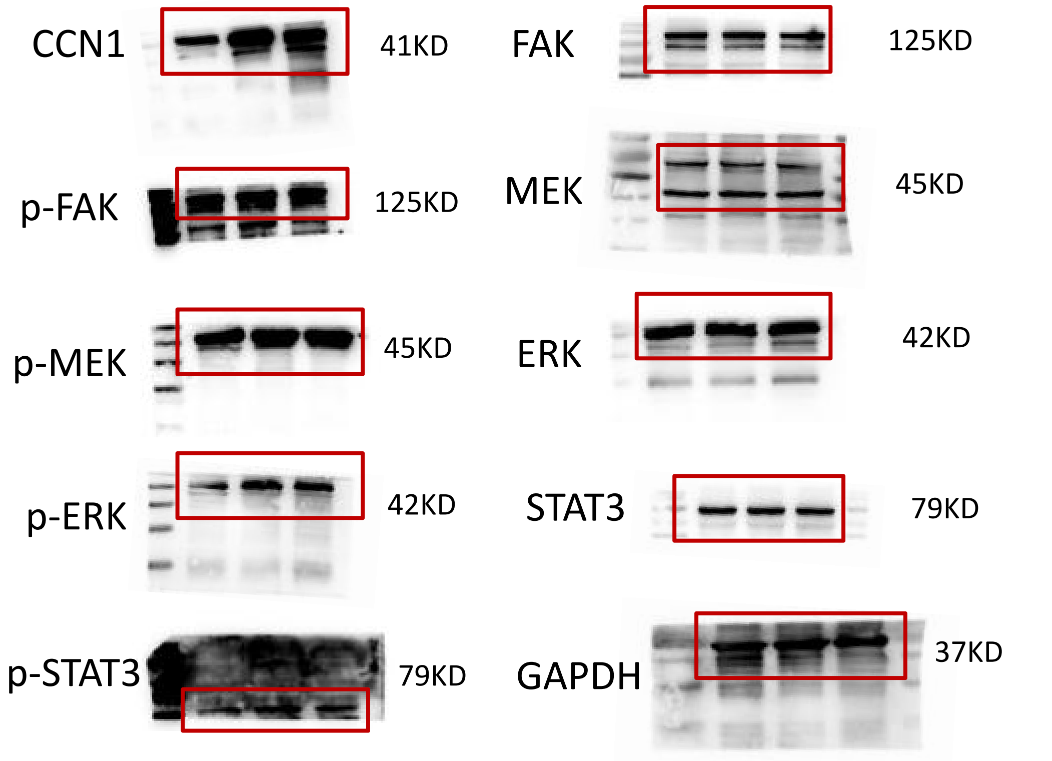


## Figure 4I

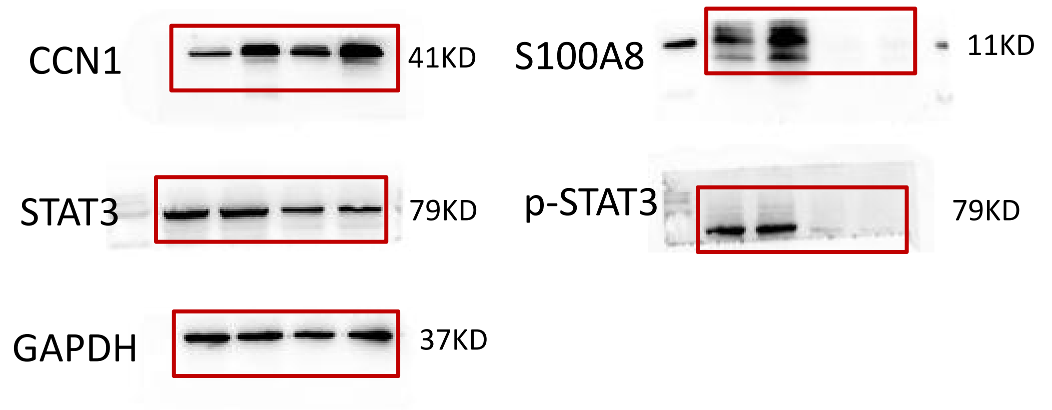


## Figure 5A

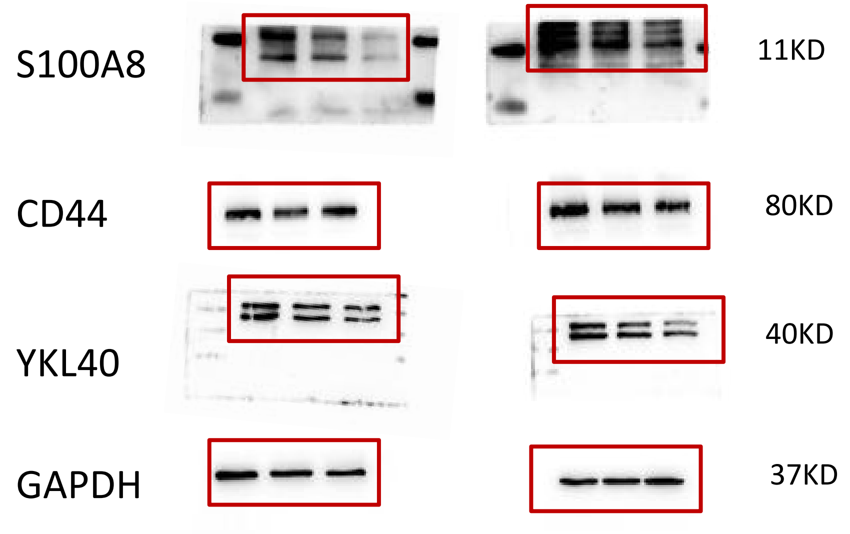

## Figure 5G

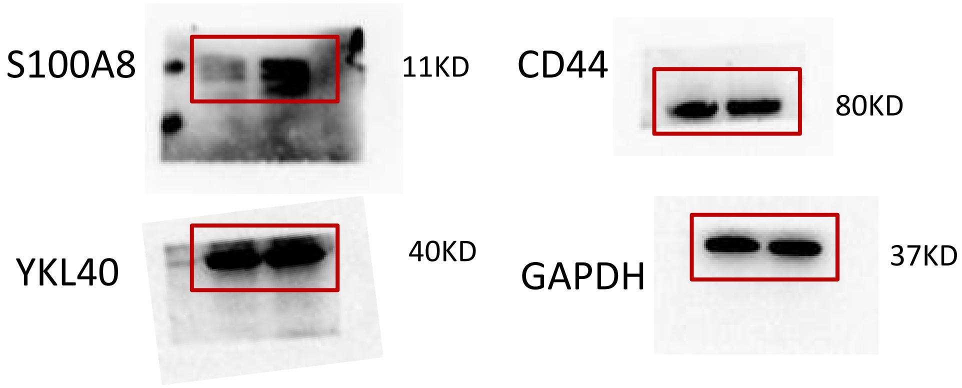


## Figure 6B

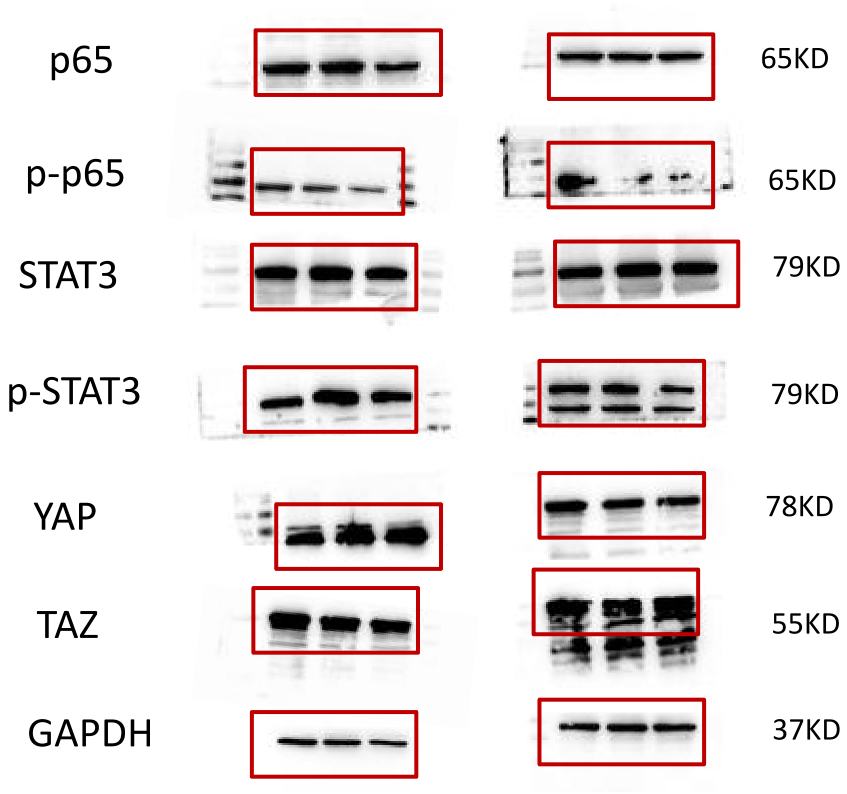


## Figure 6C

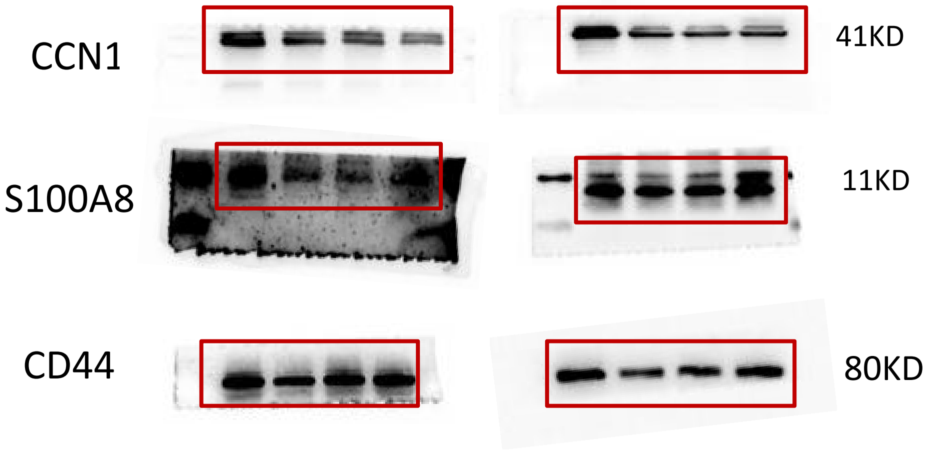

Figure 6C

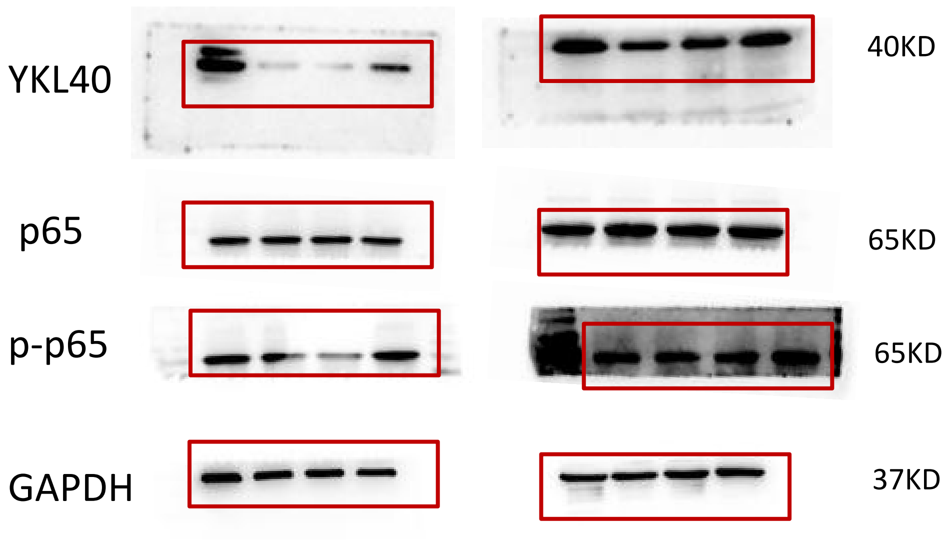


Figure 7A

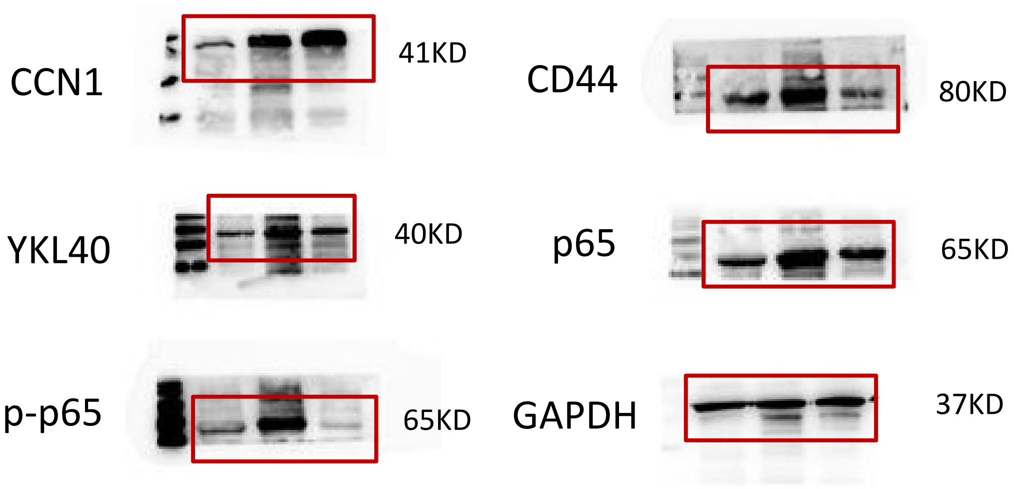


Figure S5A

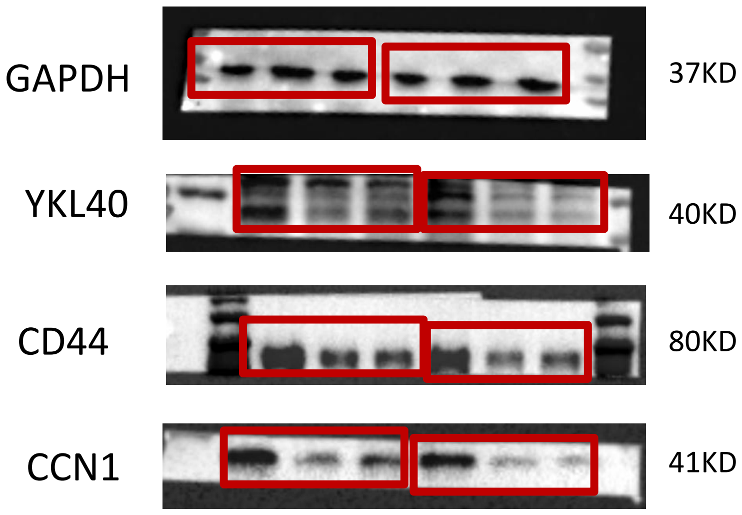

## Figure S5B

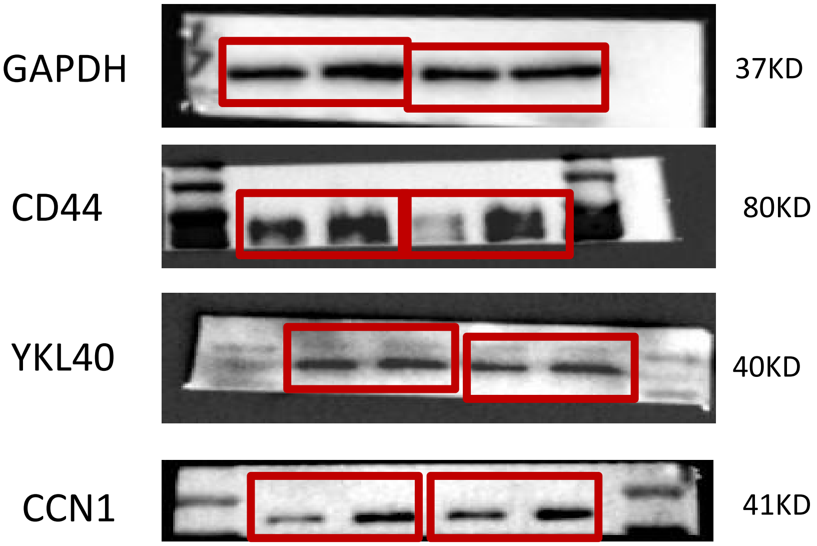


## Figure S6A

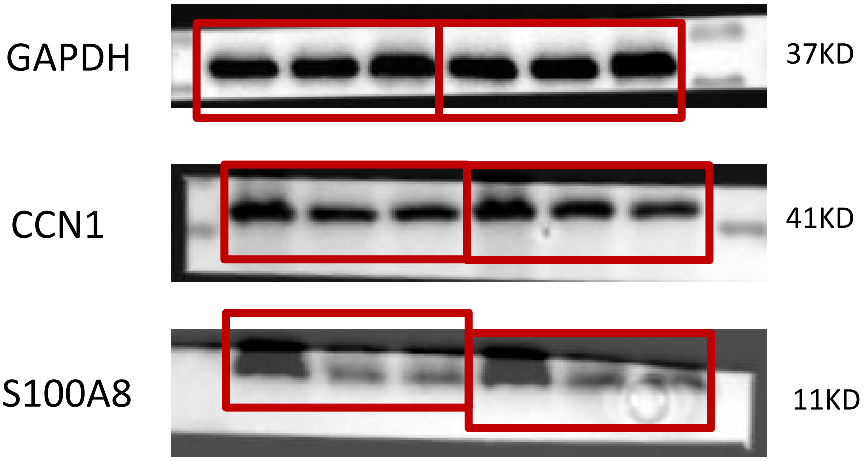


## Figure S6B

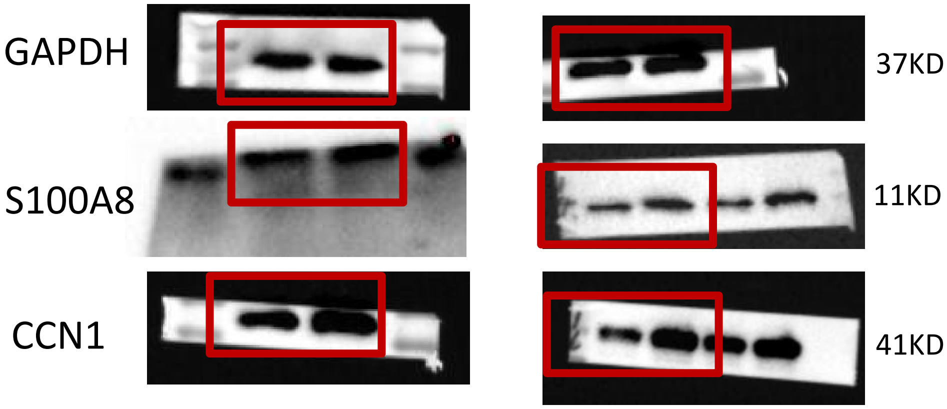

## Figure S6C

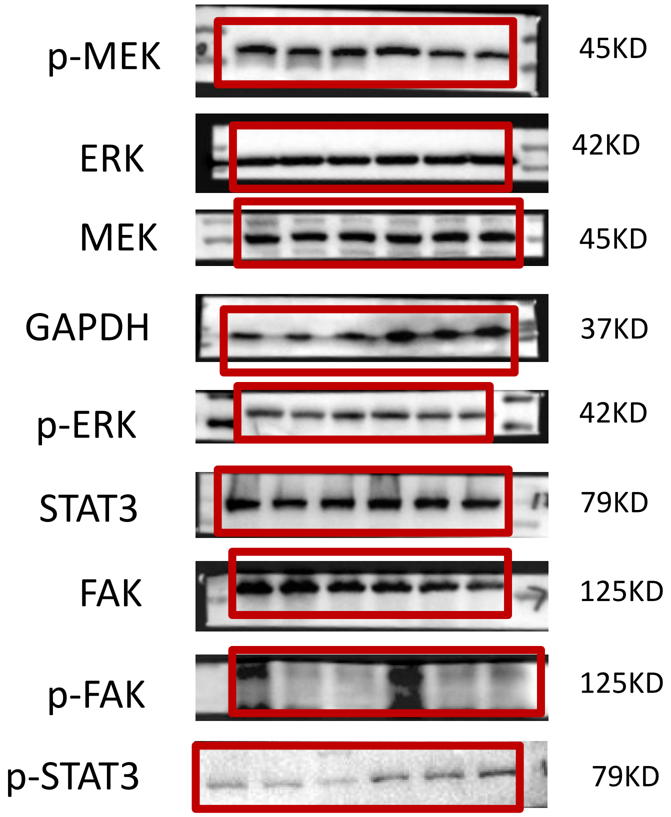


## Figure S6D

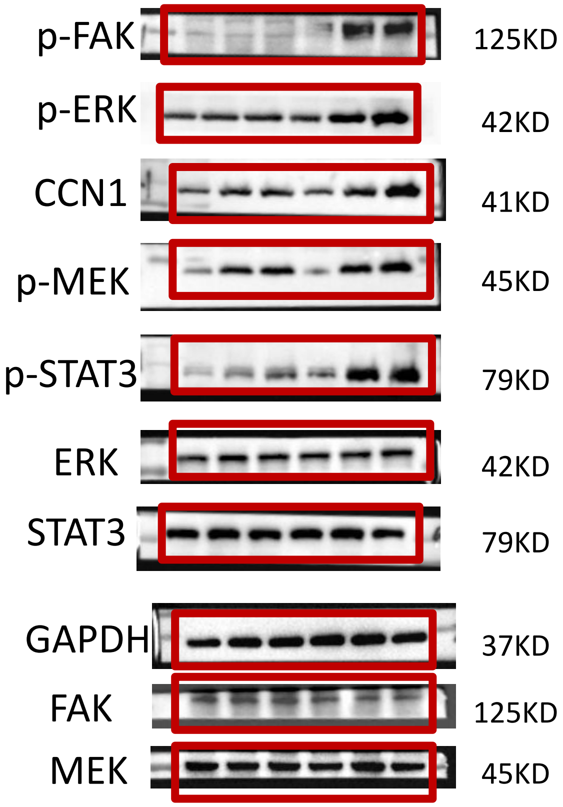

# Figure S7A

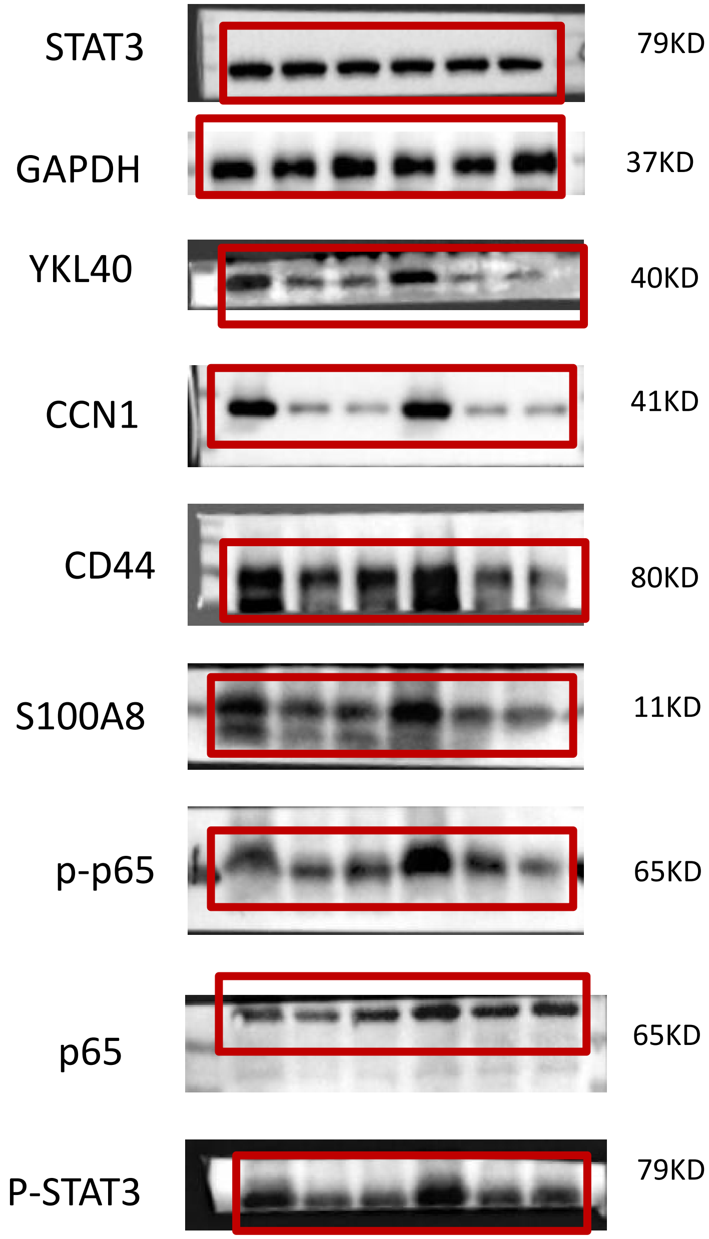
STAT3
79KD
GAPDH
37KD
YKL40
40KD
CCN1
41KD
CD44
80KD
S100A8
11KD
p-p65
65KD
p65
65KD
P-STAT3
79KD

# Figure S8A

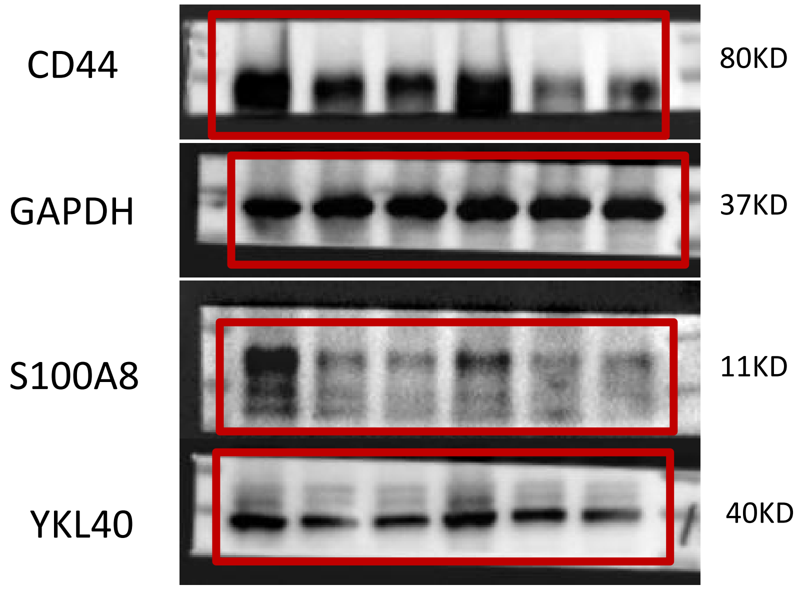


# Figure S8B

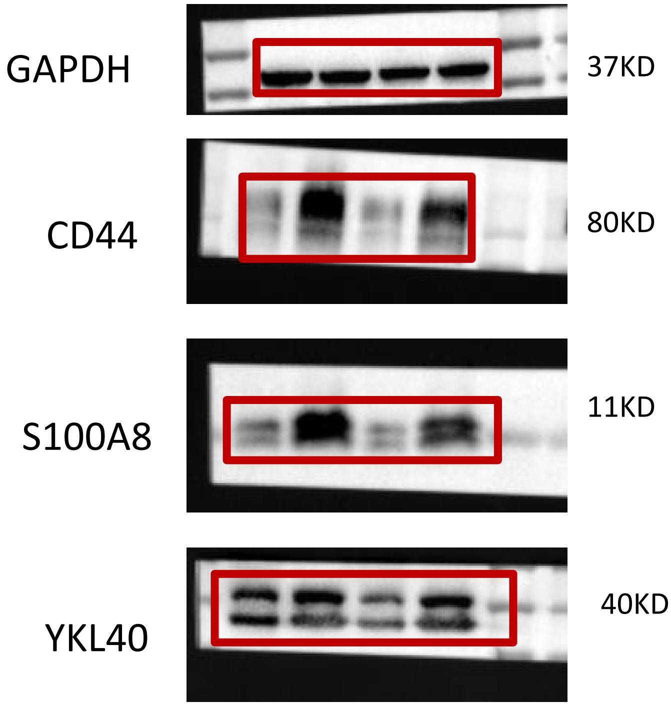

Supplement: Supplementary file 1 — Data S1. [file CNS-30-e70128-s001.pdf]
