# Supplementary material for: CCN1 Promotes Mesenchymal Phenotype Transition Through Activating NF‐κB Signaling Pathway Regulated by S100A8 in Glioma Stem Cells
Source: CNS Neurosci Ther. 2024 Dec 11;30(12):e70128. doi: 10.1111/cns.70128 (PMC11632201; doi:10.1111/cns.70128)
Supplement: Supplementary file 2 — Data S2. [file CNS-30-e70128-s002.docx]

Supplementary Materials for

CCN1 promotes mesenchymal phenotype transition through activating NF-κB signaling pathway regulated by S100A8 in glioma stem cells


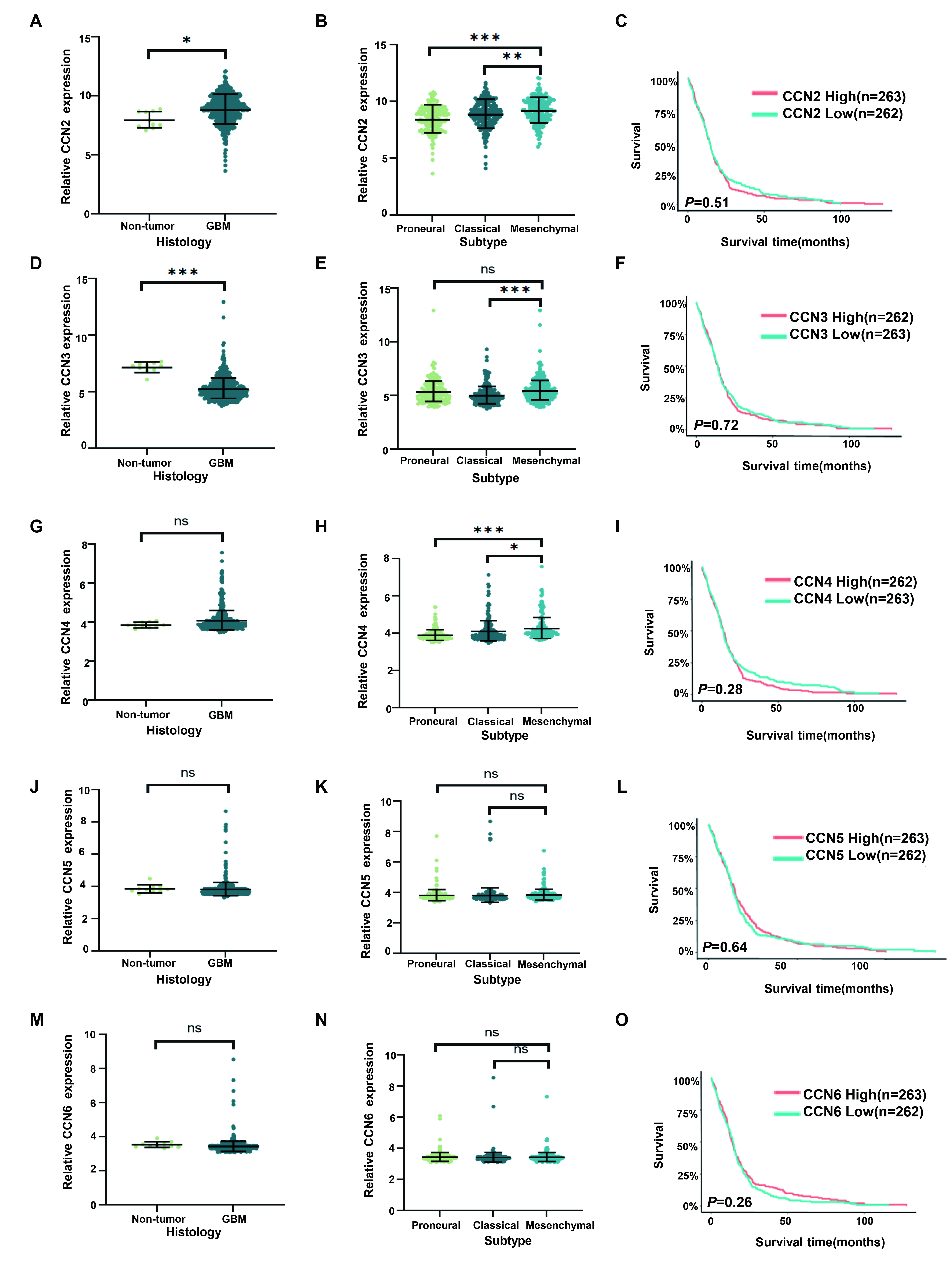


**Fig. S1.**

**The expression of CCN family members (CCN2-CCN6)was analyzed by TCGA database**

**(**A-B) The mRNA expression of CCN2 was shown according to GBM or Non-tumor and the molecular subtypes in the TCGA datasets.

**(**C) Kaplan–Meier analysis of patients with GBM with high CCN2 expression versus low CCN2 expression in the TCGA datasets.

**(**D-E) The mRNA expression of CCN3 was shown according to GBM or Non-tumor and the molecular subtypes in the TCGA datasets.

**(**F) Kaplan–Meier analysis of patients with GBM with high CCN3 expression versus low CCN3 expression in the TCGA datasets.

**(**G-H) The mRNA expression of CCN4 was shown according to GBM or Non-tumor and the molecular subtypes in the TCGA datasets.

**(**I) Kaplan–Meier analysis of patients with GBM with high CCN4 expression versus low CCN4 expression in the TCGA datasets.

**(**J-K) The mRNA expression of CCN5 was shown according to GBM or Non-tumor and the molecular subtypes in the TCGA datasets.

**(**L) Kaplan–Meier analysis of patients with GBM with high CCN5 expression versus low CCN5 expression in the TCGA datasets.

**(**M-N) The mRNA expression of CCN6 was shown according to GBM or Non-tumor and the molecular subtypes in the TCGA datasets.

**(**O) Kaplan–Meier analysis of patients with GBM with high CCN6 expression versus low CCN6 expression in the TCGA datasets.*P < 0.05, **P < 0.01, ***P < 0.001.


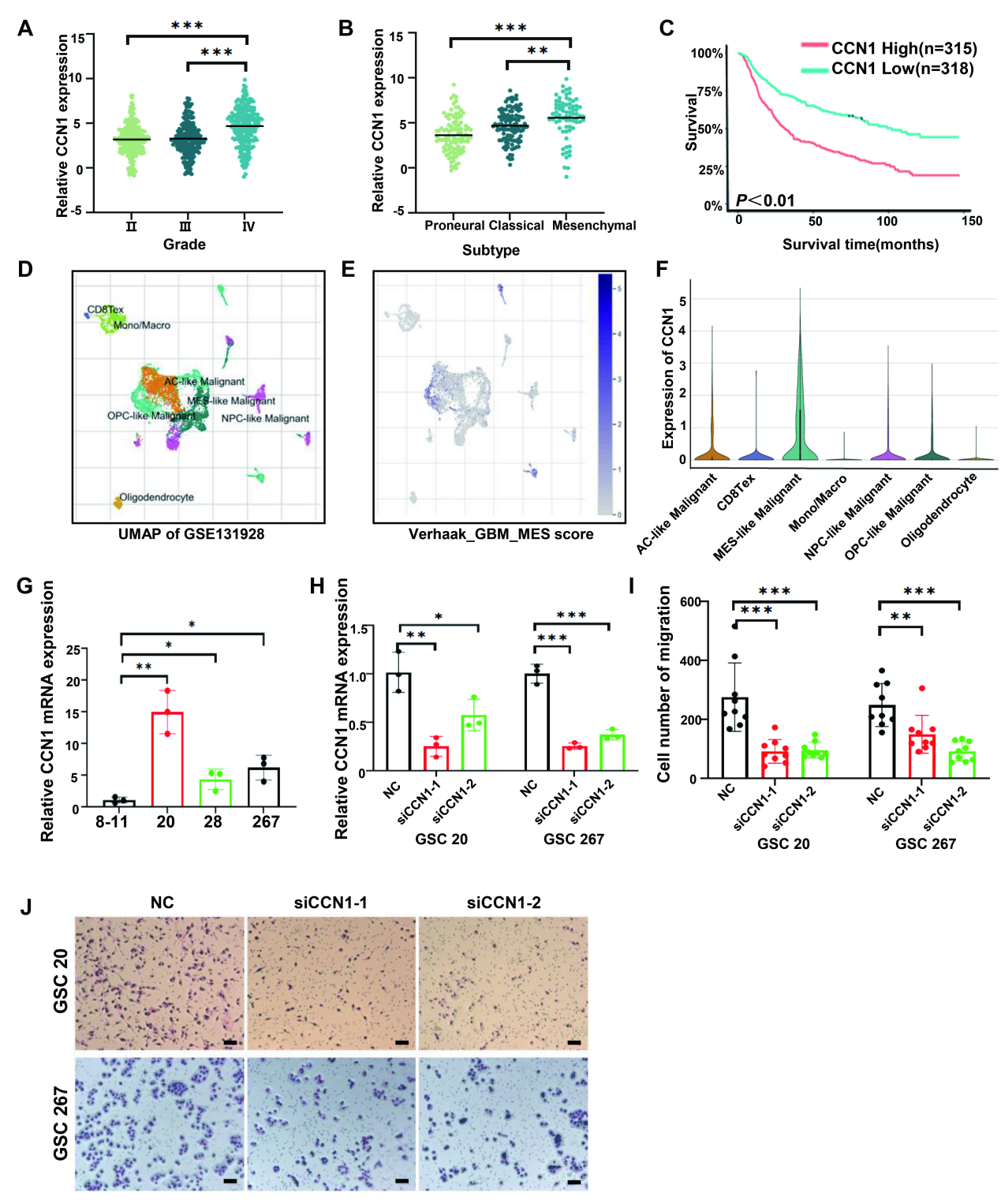


**Fig. S2.**

**CGGA database and single cell sequencing were used to analyze the expression of CCN1 and the relevant functional results of CCN1**

(A-B) The mRNA expression of CCN1 was shown according to WHO grades and the molecular subtypes in the CGGA datasets.

**(**C) Kaplan–Meier analysis of patients with glioma with high CCN1 expression versus low CCN1 expression in the CGGA datasets.

(D-F) Single-cell RNA sequencing of GSE131928 visualizing UMAP cell clusters, Verhaak_GBM_MES score and CCN1 expression.

**(**G) The expression of CCN1 in different molecular subtypes of GSCs was detected by qPCR.

**(**H)The expression of CCN1 in GSC 20 and GSC 267 after CCN1 knockdown was measured by qPCR.

(I-J) Transwell assay showed the migration of GSC 20 and GSC 267 after CCN1 knockdown. Scale bar=100μm.*P < 0.05, **P < 0.01, ***P < 0.001.


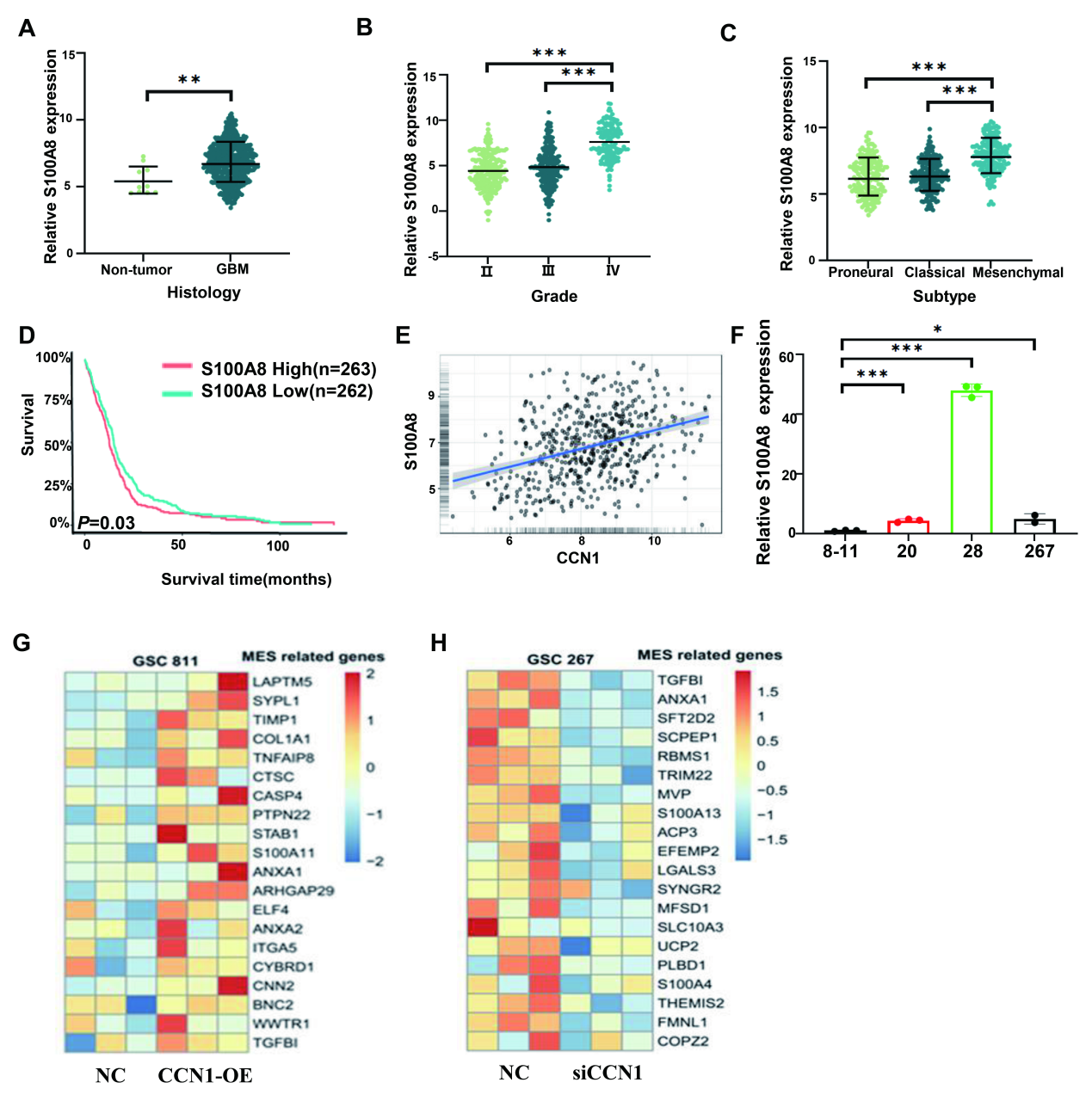


**Fig. S3.**

**TCGA database analysis of S100A8 expression and sequencing results of MES correlation analysis**

(A-C) The mRNA expression of S100A8 was shown according to GBM or Non-tumor, WHO grades and the molecular subtypes in the TCGA datasets.

(D) Kaplan–Meier analysis of patients with GBM with high S100A8 expression versus low S100A8 expression in the TCGA datasets.

(E) TCGA dataset analysis showed a positive correlation between CCN1 and S100A8 transcription levels.

(F) The expression of S100A8 in different molecular subtypes of GSCs was detected by qPCR.

(G-H) Heatmaps of MES phenotype correlation genes analysis after knockdown and overexpression of CCN1.*P < 0.05, **P < 0.01, ***P < 0.001.


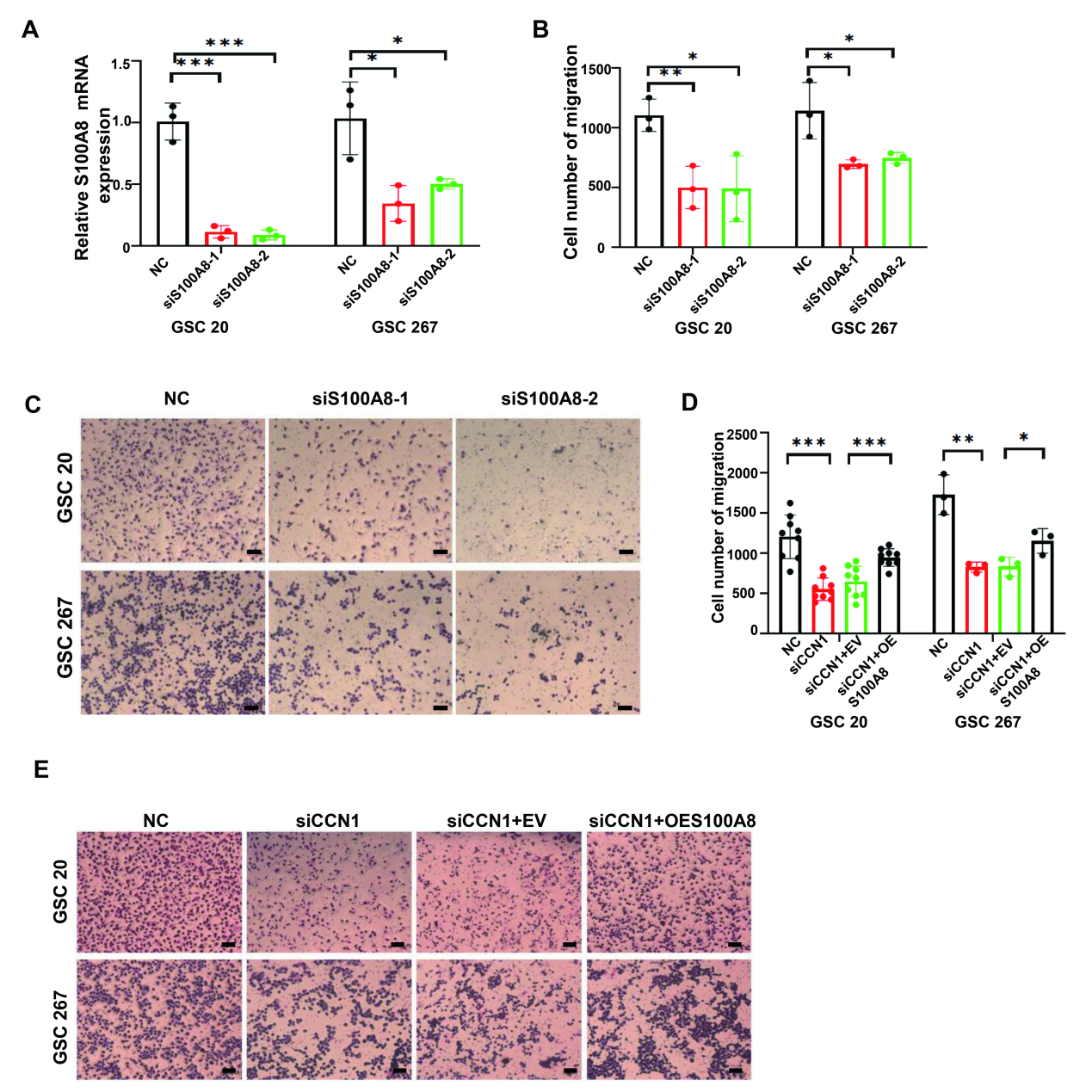


**Fig. S4.**

**Experimental results of S100A8 function**

(A) The expression of S100A8 in GSC 20 and GSC 267 after S100A8 knockdown was measured by qPCR.

(B-C) Transwell assay showed the migration of GSC 20 and GSC 267 after S100A8 knockdown. Scale bar=100μm.

(D-E) Transwell assay showed that the migration ability of GSC 20 and GSC 267 were restored after S100A8 reexpression. Scale bar=100μm.*P < 0.05, **P < 0.01, ***P < 0.001.


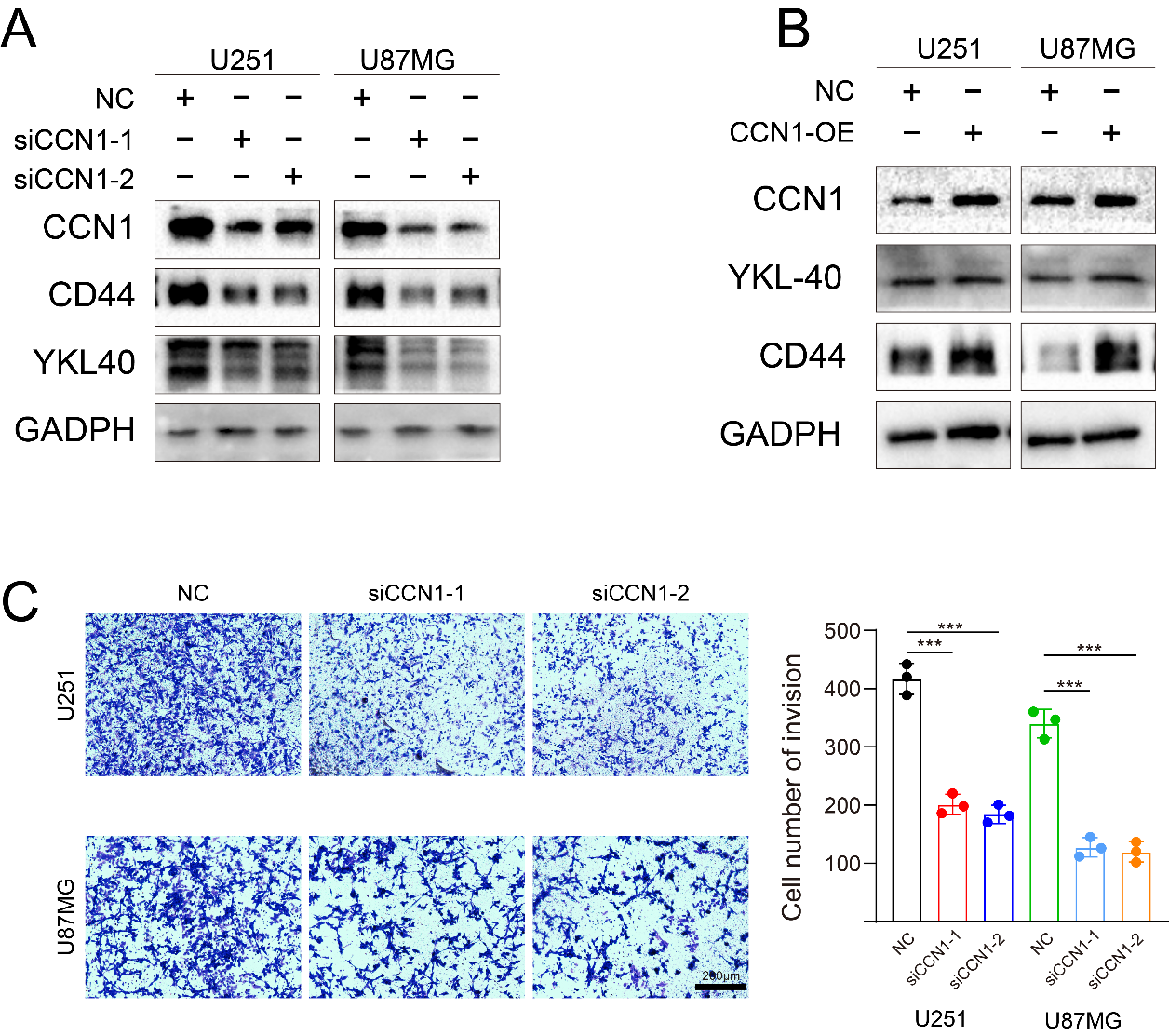


**Fig. S5.**

**CCN1 knockdown in GBM cell lines**

(A-B) Knockdown of CCN1 expression significantly inhibited the expression of MES subtype markers (CD44 and YKL40) in GBM cell lines, while overexpression of CCN1 exhibited the opposite effect.

(C) Transwell assay showed the invasion of GBM cell lines (U251 and U87MG) after CCN1 knockdown. Scale bar=200μm. *P < 0.05, **P < 0.01, ***P < 0.001.


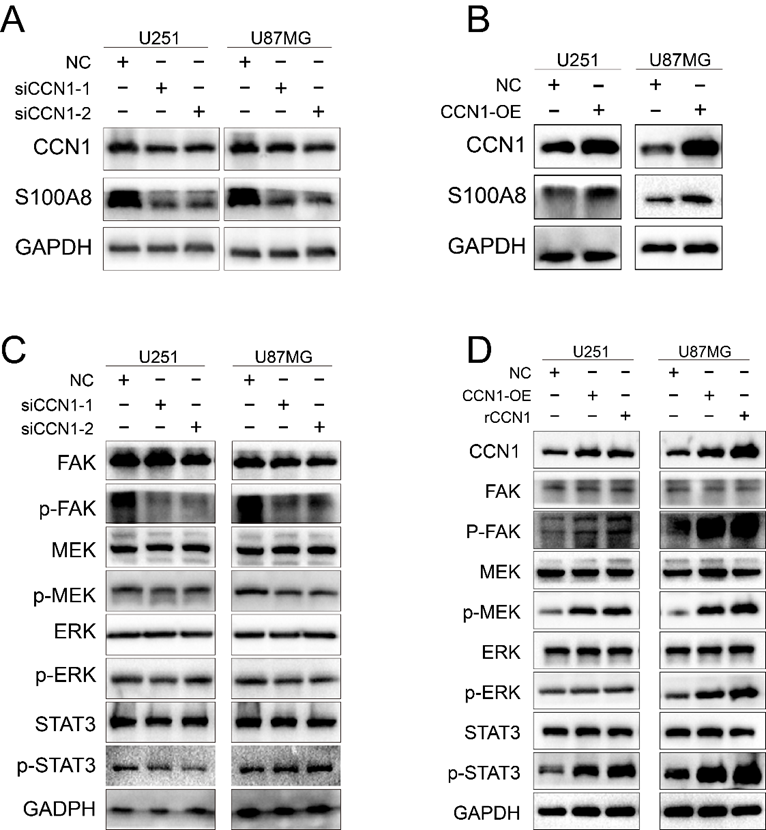


**Fig. S6.**

**CCN1 regulates the expression of S100A8 via FAK-STAT3 signaling in GBM cell lines**

(A-B) The effect of CCN1 knockdown or overexpression on the expression of S100A8 was examined in GBM cell lines.

(C-D) The effect of CCN1 knockdown or overexpression on the expression of signaling pathway proteins (FAK, MEK, ERK, STAT3) were examined in GBM cell lines.


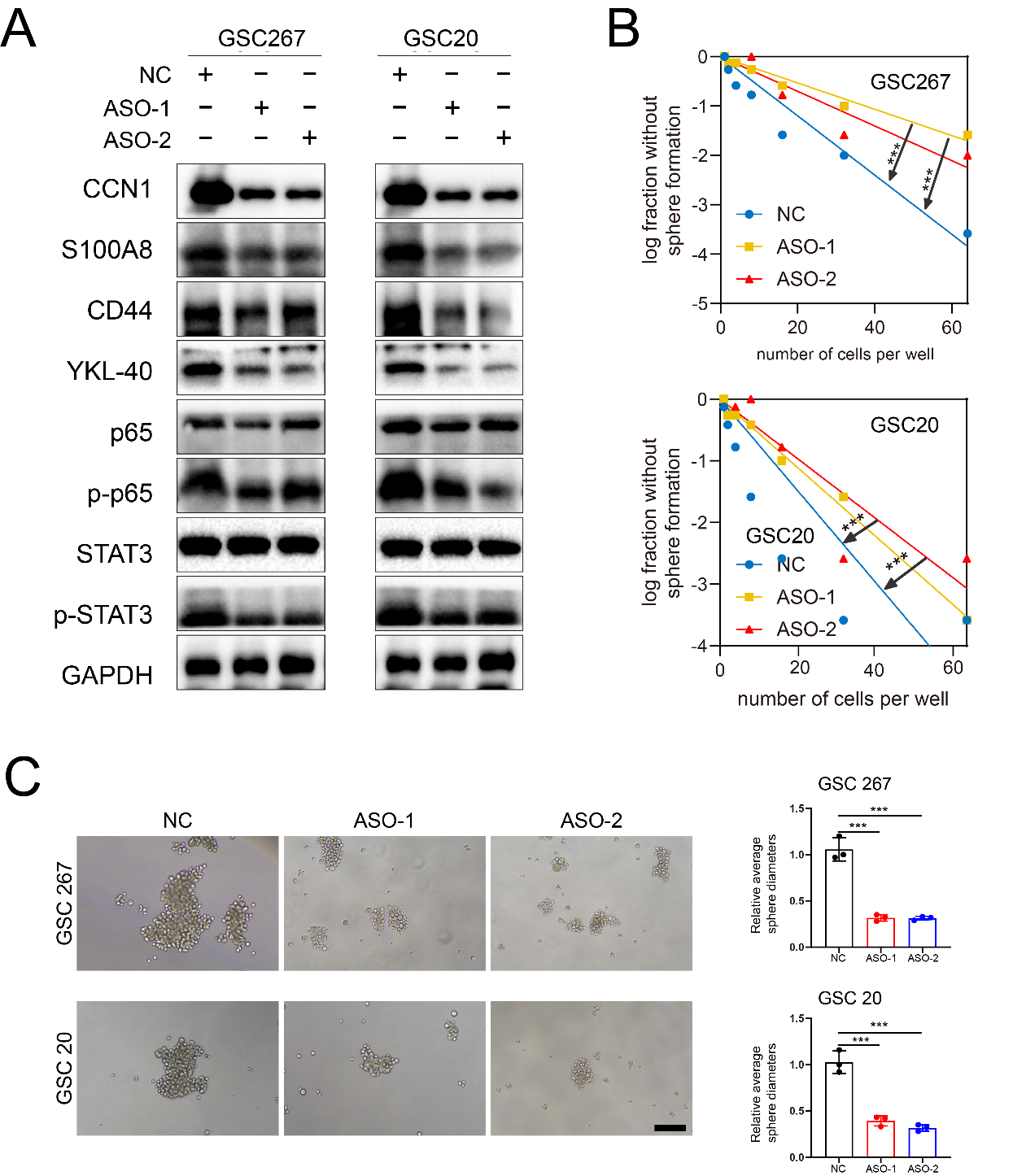


**Fig. S7.**

**The effect of ASO targeting CCN1 on GSCs**

(A) The effect of CCN1 inhibitor ASO on the expression of GBM subtype markers and key pathway proteins were examined in GSCs.

(B) The effect of CCN1 inhibitor ASO on the GSCs were evaluated by limiting dilution assays.

(C) Representative images and quantification of tumor sphere formation of GSCs treated with CCN1 inhibitor ASO. Scale bar, 100 μm. *P < 0.05, **P < 0.01, ***P < 0.001.


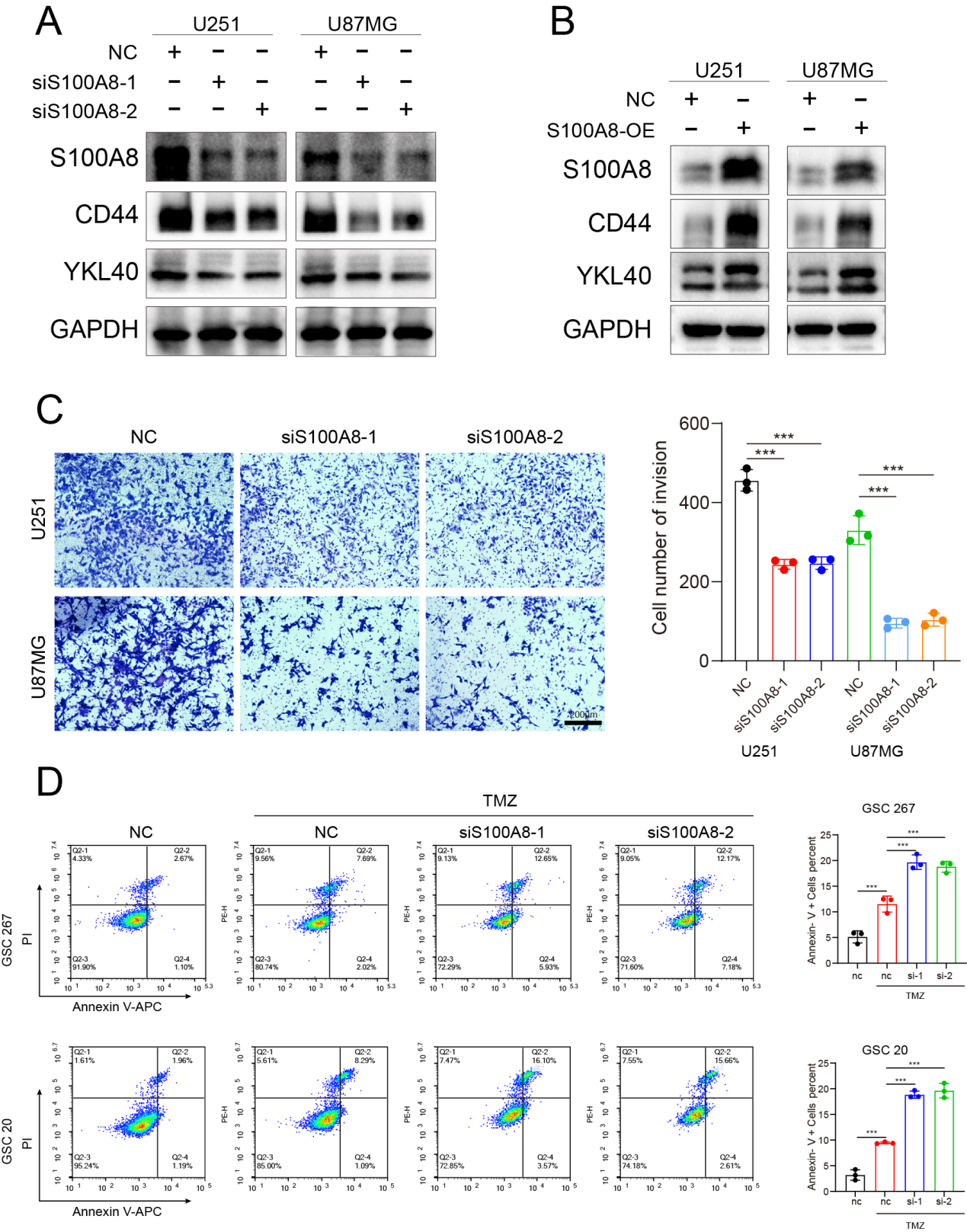


**Fig. S8.**

**S100A8 knockdown in GBM cell lines**

(A-B) The expression of S100A8 and MES phenotype markers in GBM cell lines after S100A8 knockdown or overexpression were measured by western blotting.

(C) Transwell assay showed the invasion of GBM cell lines after S100A8 knockdown. Scale bar=200μm.

(D)Flow cytometric analysis showing the impact of S100A8 knockdown on the apoptosis of GSCs treated with TMZ (500μM). *P < 0.05, **P < 0.01, ***P < 0.001.

**Supplementary Table1**

**Sequences of the siRNA**

| Name | species | Forward (5' -> 3') | Reverse (5' -> 3') |
| --- | --- | --- | --- |
| siCCN1-1 | human | CCUGCAAAUGCAACUACAATT | UUGUAGUUGCAUUUGCAGGTT |
| siCCN1-2 | human | GCAUCCUAUACAACCCUUUTT | AAAGGGUUGUAUAGGAUGCTT |
| siS100A8-1 | human | CUAUCAUCGACGUCUACCATT | UGGUAGACGUCGAUGAUAGTT |
| siS100A8-2 | human | CUGAAGAAAUUGCUAGAGATT | UCUCUAGCAAUUUCUUCAGTT |
| si-NC | human | UUCUCCGAACGUGUCACGUTT | ACGUGACACGUUCGGAGAATT |

**Supplementary Table2**

**CCN1 lentivirus sequence**

| Name | Sequence (5’-3’) |
| --- | --- |
| LV16-shCCN1 | GCATCCTATACAACCCTTT |
| LV16NC | TTCTCCGAACGTGTCACGT |
| CCN1-OE | From CCDS706.1（*Homo sapiens*） |

**Supplementary Table3**

**Primer sequences for Real-time PCR**

| Gene | species | Forward (5' -> 3') | Reverse (5' -> 3') |
| --- | --- | --- | --- |
| CCN1 | human | CAGGACTGTGAAGATGCGGT | GCCTGTAGAAGGGAAACGCT |
| S100A8 | human | CTAGAGACCGAGTGTCCTCAGTAT | CCACGCCCATCTTTATCACC |
| GAPDH | human | GCACCGTCAAGGCTGAGAAC | TGGTGAAGACGCCAGTGGA |

**Supplementary Table4**

**Antibody for Western blots**

| Antibody | Manufacturer | Cat. # | Dilution |
| --- | --- | --- | --- |
| GAPDH | Cell Signaling Technology,USA | 5174 | 1:1000 |
| CCN1 | Cell Signaling Technology,USA | 14479 | 1:1000 |
| CD44 | Cell Signaling Technology,USA | 37259 | 1:1000 |
| YKL40 | Cell Signaling Technology,USA | 47066 | 1:1000 |
| Phospho-NF-κB p65 | Cell Signaling Technology,USA | 3033 | 1:1000 |
| NF-κB p65 | Cell Signaling Technology,USA | 8242 | 1:1000 |
| Phospho-STAT3 | Cell Signaling Technology,USA | 9145 | 1:1000 |
| STAT3 | Cell Signaling Technology,USA | 9139 | 1:1000 |
| YAP/TAZ | Cell Signaling Technology,USA | 8418 | 1:1000 |
| S100A8 | Abcam,UK | ab92331 | 1:1000 |
| Antibody | Manufacturer | Cat. # | Dilution |
| FAK | Cell Signaling Technology,USA | 3285 | 1:1000 |
| p-FAK | Cell Signaling Technology,USA | 3283 | 1:1000 |
| MEK | Affinity Bioscience,USA | AF6385 | 1:1000 |
| p-MEK | Affinity Bioscience,USA | AF8035 | 1:1000 |
| ERK | Cell Signaling Technology,USA | 4695 | 1:1000 |
| p-ERK | Cell Signaling Technology,USA | 4370 | 1:1000 |

**Supplementary Table5**

**Catalogue number of all reagents/kits**

| Reagents/kits | Cat. # |
| --- | --- |
| DMEM/F-12, GlutaMAX™ additive | 10565018 |
| B-27™ Additive (50X), serum free | 17504044 |
| Recombinant Human EGF Protein | 236-EG |
| Recombinant Human FGF basic Protein | 233-FB |
| Accutase® solution | A6964-500ML |
| 10× polylysine | P2100 |
| Serum-free cell freeze | C40100 |
| Annexin V-FITC/PI double staining apoptosis detection kit | BB-4101 |
| Lipofectamine™ 3000 transfection reagent | L3000075 |
| JSH-23 | HY-13982 |
| Angoline | TN6739 |
| Recombinant Human Cyr61/CCN1 Protein | 4055-CR-050 |
| RNA Fast 200 extraction kit | 220011 |
| Evo M-MLV reverse transcription kit | AG11754 |
| SYBR Green Pro Taq HS premixed qPCR reagent | AG11759 |
| ECL luminescent solution | PE0010 |
| Luciferin potassium salt | 50227 |
| TMZ | HY-17364 |
